# CRISPR-mediated rapid arming of poxvirus vectors enables facile generation of the novel immunotherapeutic STINGPOX

**DOI:** 10.3389/fimmu.2022.1050250

**Published:** 2023-01-13

**Authors:** Jack T. Whelan, Ragunath Singaravelu, Fuan Wang, Adrian Pelin, Levi A. Tamming, Giuseppe Pugliese, Nikolas T. Martin, Mathieu J. F. Crupi, Julia Petryk, Bradley Austin, Xiaohong He, Ricardo Marius, Jessie Duong, Carter Jones, Emily E. F. Fekete, Nouf Alluqmani, Andrew Chen, Stephen Boulton, Michael S. Huh, Matt Y. Tang, Zaid Taha, Elena Scut, Jean-Simon Diallo, Taha Azad, Brian D. Lichty, Carolina S. Ilkow, John C. Bell

**Affiliations:** ^1^ Department of Biochemistry, Microbiology and Immunology, University of Ottawa, Ottawa, ON, Canada; ^2^ Centre for Innovation Cancer Therapeutics, Ottawa Hospital Research Institute, Ottawa, ON, Canada; ^3^ Public Health Agency of Canada, Ottawa, ON, Canada; ^4^ McMaster Immunology Research Centre, Department of Medicine, McMaster University, Hamilton, ON, Canada; ^5^ MG DeGroote Institute for Infectious Disease Research, McMaster University, Hamilton, ON, Canada

**Keywords:** CRISPR/Cas9 (clustered regularly interspaced short palindromic repeats/CRISPR-associated protein 9), vaccinia virus (VACV), STING agonist, poxvirus, oncolytic virus

## Abstract

Poxvirus vectors represent versatile modalities for engineering novel vaccines and cancer immunotherapies. In addition to their oncolytic capacity and immunogenic influence, they can be readily engineered to express multiple large transgenes. However, the integration of multiple payloads into poxvirus genomes by traditional recombination-based approaches can be highly inefficient, time-consuming and cumbersome. Herein, we describe a simple, cost-effective approach to rapidly generate and purify a poxvirus vector with multiple transgenes. By utilizing a simple, modular CRISPR/Cas9 assisted-recombinant vaccinia virus engineering (CARVE) system, we demonstrate generation of a recombinant vaccinia virus expressing three distinct transgenes at three different loci in less than 1 week. We apply CARVE to rapidly generate a novel immunogenic vaccinia virus vector, which expresses a bacterial diadenylate cyclase. This novel vector, STINGPOX, produces cyclic di-AMP, a STING agonist, which drives IFN signaling critical to the anti-tumor immune response. We demonstrate that STINGPOX can drive IFN signaling in primary human cancer tissue explants. Using an immunocompetent murine colon cancer model, we demonstrate that intratumoral administration of STINGPOX in combination with checkpoint inhibitor, anti-PD1, promotes survival post-tumour challenge. These data demonstrate the utility of CRISPR/Cas9 in the rapid arming of poxvirus vectors with therapeutic payloads to create novel immunotherapies.

## Introduction

Since its early use in the smallpox eradication campaign, vaccinia virus (VACV) has been established as a prototypical poxvirus for engineering vaccine vectors to combat cancer and infectious diseases (1). VACV-based vectors are versatile, with applications in immunization, gene therapy, and oncolytic virotherapy. Several of the intrinsic properties of VACV make it an ideal vector, including a large coding capacity for expression of therapeutic transgenes, a cytoplasmic life cycle which limits risks of integration into host genome, and the ability to manipulate viral tropism through deletion of host-range and virulence factors (2).

Despite its widespread use as a vector, the standard engineering approaches used to attenuate the viral genome or encode transgenes remain largely unchanged from foundational protocols developed decades ago (3, 4). The standard engineering approaches rely primarily on the homologous recombination of VACV. The recombination is mediated by virally expressed factors, due to the isolation of VACV replication complexes in the cytoplasm, away from nuclear host factors (reviewed in (5). VACV’s recombination capacity is required for productive replication of the large double stranded DNA poxvirus genome. The key viral factors for recombination include the viral polymerase (E9L), a single-stranded binding protein (I3L), Holliday junction resolvase (A22R), and DNA ligase (A48R) (6–10). Early work demonstrated that providing a recombination template in the presence of replicating VACV was sufficient to yield genetically modified viruses (3). This work has served as the basis for the traditional approach for recombinant VACV rescue, which involves the infection of cells with a parental virus and simultaneous delivery of a donor template for homologous recombination, usually in the form of plasmid or PCR product. These homologous recombination templates generally include a transgene, a marker for selection, and flanking homology domains targeting the insertion site. Recombination rates have been reported from 0.1 to 5%; hence, recombinant viral species must be purified by collecting clonal viral populations from single plaques (known as plaque picking) or other isolation methods (e.g. flow cytometry) (11). The methods can be quite time consuming, require the use of markers (e.g. fluorophore, antibiotic resistance marker) and necessitate several rounds of purification approaches to isolate clonal viral preparations. These technical obstacles are further compounded when aiming to rescue viruses with multiple target mutations or multiple therapeutic transgenes in parallel. The implementation of poxvirus recombination and purification strategies circumventing these limitations would enable the development of high-throughput screening approaches to compare and contrast potential therapeutic transgenes.

An attractive tool in the development of higher efficiency engineering strategies for VACV is CRISPR-Cas9 technologies. CRISPR-Cas9 systems have been adapted to increase the rate of homologous recombination in various organisms (bacteria, mammals, etc.) as well as viruses (herpesviruses, adenoviruses, poxviruses etc.) (12–15). These viral engineering applications rely on the specific cleavage of the viral genome followed by homologous recombination template (HRT) mediated repair. Strategic application of this gene-editing strategy to the VACV genome may not only improve recombination efficiency, but can also serve as form of negative selection against the unedited viral genome through the use of parental strain-targeted Cas9 cleavage activity (15).

There have been numerous efforts to enhance VACV-based vectors’ immunological influence by encoding immunostimulatory payloads in the viral genome in both the context of vaccine and oncolytic virus development (2). However, STING agonists represent one class of payload which has yet to be examined as a transgene in a poxvirus vector. STING (STING1) is an endoplasmic reticulum-localized receptor of cyclic dinucleotides (16). Recognition of cyclic dinucleotides (CDNs) produced as secondary messengers by bacteria, including cyclic di-AMP or di-GMP, or the enzyme cyclic GMP-AMP synthase, a key innate immune sensor of intracellular DNA, induces STING phosphorylation and dimerization (17). This yields activation of STING signaling, which results in a downstream phosphorylation cascade of key signaling components (e.g. TBK1) which regulate type I interferon (IFN) and inflammatory cytokine production (18).

Recent studies have demonstrated that direct activation of STING signaling in the tumor microenvironment yields a systemic anti-cancer response (19). While pre-clinical studies have shown great promise for pharmaceutical-based natural and synthetic STING agonists in the treatment of cancer, limited success has been achieved in clinical trials. Potential barriers to clinical success include membrane permeability, adverse effects, such as cytokines storms, and poor stability – restricting the potential systemic delivery applications (20, 21). Hence, to-date, the majority of clinical trials investigating STING agonists have been limited to indications utilizing intratumoral administration (20). The capacity of an oncolytic VACV-based vector to generate STING agonists *in situ* in an oncoselective fashion should circumvent these limitations, enabling effective systemic delivery of STING agonists to the cancer cell (22).

Herein we describe a CRISPR/Cas9 based approach for the rapid generation of recombinant VACV expressing multiple payloads. Through the use of a stable cell line expressing Cas9 and transfection of single guide RNAs (sgRNAs) targeting desired loci, we develop a simple and highly modular strategy for viral gene deletion and transgene insertion at multiple loci. The process enables purification of recombinant VACV expressing three transgenes inserted at three different loci within 1 week from initial infection/transfection. As a proof-of-concept for this platform, we leveraged our accelerated recombinant VACV generation methodology to generate a novel immunogenic VACV vector-based oncolytic virus delivering a bacterial cyclase to synthesize STING agonizing cyclic dinucleotides in the tumor microenvironment.

## Materials and methods

### Cell lines, plasmids, and viruses

pLentiCas9 Blast was a gift from Feng Zhang (Addgene plasmid # 52962). pLenti CMV/TO Puro empty (w175-1) was a gift from Eric Campeau & Paul Kaufman (Addgene plasmid # 17482).

pLenti-sgRNA Blast was a gift from Brett Stinger (Addgene plasmid #104993). U2OS (ATCC; HTB-96), HT29 (ATCC; HTB-33), 293T (ATCC, CRL-3216) and HeLa cells (ATCC; CCL-2) were grown in DMEM supplemented with untreated 10% fetal bovine serum (FBS). B16-OVA cells were a kind gift from Dr. Rebecca Auer (Ottawa Hospital Research Institute, Canada). THP1-Blue ISG cells (thp-isg), THP1-Dual (thpd-nfis), THP1-Dual KO-STING (thpd-kostg), THP1-Dual KO-cGAS (thpd-kocgas) and 293-Dual hSTING-R232 cells (293d-r232) were purchased from InvivoGen and grown as per manufacturer’s protocols. Of note, THP1 cells have the STING HAQ allele found in 20% of population whereas R232 is the STING “WT” allele found in ~60% of population (23). Modified vaccinia Ankara (MVA) and the BHK-21 cells used to propagate MVA were obtained from ATCC (VR-1508 and CCL-10, respectively). BHK-21 cells were cultured in DMEM supplemented with 10% FBS. Dr. David Evans (University of Alberta, Canada) generously provided recombinant Tian Tan viruses and a clone with 99% similarity to Tian Tan WT (DTH14) was selected for use in this study (24). Copenhagen strain VACV was a generous gift from Dr. Grant McFadden (Arizona State University, US). Western Reserve strain VACV was purchased from ATCC (VR-1354). OncoVACV is a novel attenuated VACV vector that will be described in a separate publication (25).

### U2OS-Cas9 and U2OS-Cas9-NLS cell line generation

Lentivector expressing Cas9 without nuclear localization sequence was generated by subcloning Cas9 without NLS from lenti-Cas9-blast (26), using primers described in **Supplementary Table 1**, into pLenti CMV/TO Puro (27). Restriction cloning was performed using BamHI/XbaI restriction enzymes. Lentivirus was generated by transfecting use Lenti-X 293T cell line (Takarabio) by transfecting cells with pCMV-TO Puro-Cas9 or pLenti-Cas9-Blast (contains NLS), pSPAX2, and pMD.2 (3:2:1 ratio) using Lipofectamine 2000 (2 µL/1ug DNA). Supernatants were collected approximately 48 and 72h hours after transfection and passed through a 0.45-μm filter and added to cells. Appropriate antibiotic cell selection was performed to isolate stably transduced cell lines. Clonal selection of U2OS-Cas9 cells was performed by limiting dilution and immunoblot analyses was performed to identify clones with the highest level of Cas9 expression (refer to **Supplementary Figure 1**). U2OS-Cas9 cells were maintained under puromycin selection (1 μg/mL).

### Guide RNAs

Synthetic guide RNAs were purchased from Synthego (Redwood, CA, USA). Guides were designed with the highest “on-target” score that were compatible with the desired deletion based on the approach described by Doench et al. (2016) (28). Guide RNA sequences are listed in **Supplementary Table 1**. For initial validation of the CRAVE system, lentivirus was used for sgRNA delivery. Lenti sgRNA blast (29) was used to clone sgB8R and a non-targeting control (NTC) guide using oligonucleotide sequences provided in **Supplementary Table 1** using previously described protocol (26).

### Generation of B8R-targeted donor plasmid encoding GFP

To facilitate convenient assessment of recombination efficacy, the B8R coding determining sequence (CDS) was to be disrupted by successful recombination and the coding sequence of EGFP was to be inserted under the control of the VACV B8R promoter, aligning with the start codon of the B8R CDS. A donor plasmid for insertion in the B8R loci was constructed with ~500 bp homology arms (GenBank Accession: M35027.1, 5’HR 169079…169596, 3’HR 169600…170258) using the standard cloning vector pUC19 as a backbone. The plasmid was generated in a one-step cloning approach using the NEBuilder^®^ HiFi DNA Assembly Kit (New England Biolabs). This cloning Kit utilizes homologous DNA overhangs of 15-30 bp which can be added by overhanging PCR primers, facilitating seamless DNA assembly. In brief, each plasmid was assembled by fusing four fragments together: the bacterial vector backbone derived from pUC19, the 5’ homology arm, the EGFP CDS, and the 3’ homology arm. Each fragment was generated by standard PCR using primers described in **Supplementary Table 1**.

Homologous recombination templates were produced using PCR facilitated by CloneAmp HiFi PCR Premix (Takara Bio) and primers B8R_HR_F and B8R_HR_R (described in **Supplementary Table 1**) according to the manufacturer’s guidelines. Resulting DNA was isolated using Nucleospin Gel and PCR Clean-up kit (Takara Bio).

### Generation of I4L-targeted donor plasmid encoding mCherry

Geneblock designed with ~500 bp homology arms specific to the I4L loci (GenBank Accession: M35027.1, 5’HR 64640…65100, 3’HR 67397…67841) flanking a multiple cloning site upstream of an mCherry coding sequence was constructed by Integrated DNA Technologies. This was inserted into a pUC57-Kan cloning vector following AgeI and NheI cleavage of the backbone and Geneblock and ligation by T4 DNA ligase (New England Biotechnologies) following manufacturer protocol.

Homologous recombination templates were produced using PCR facilitated by CloneAmp HiFi PCR Premix (Takara Bio) and primers I4L_HR_F and I4L_HR_R (refer to **Supplementary Table 1**) according to the manufacturer’s guidelines. Resulting DNA was isolated using Nucleospin Gel and PCR Clean-up kit (Takara Bio).

### Generation of J2R and A46R homologous recombination template

Geneblock designed with ~500 bp homology arms specific to the J2R loci (GenBank Accession: M35027.1, 5’HR 83641…84130, 3’HR 84156…84655) flanking a multiple cloning site upstream of an mCherry coding sequence was constructed by Integrated DNA Technologies. A second geneblock designed with ~500 bp homology arms specific to the A46R loci (GenBank Accession: M35027.1, 5’HR 151658…152157, 3’HR 152848…153347) flanking a multiple cloning site upstream of an iRFP720 coding sequence was constructed by Integrated DNA Technologies. Homologous recombination templates were produced using PCR facilitated by CloneAmp HiFi PCR Premix (Takara Bio) and primer pairs J2R_HR_F and J2R_HR_R, and A46R_HR_F and A46R_HR_R (refer to **Supplementary Table 1**) according to the manufacturer’s guidelines. Resulting DNA was isolated using Nucleospin Gel and PCR Clean-up kit (Takara Bio).

### CRISPR assisted recombinant VACV engineering

U2OS-Cas9 cells were infected at 70-80% confluency in 24-well plates (MOI = 0.1) for 2 hours. Media was changed to OptiMEM (ThermoFisher) and cells were transfected with 500 ng homologous recombination template and 1.3 μL of 3 μM sgRNA stock (Synthego) targeting the locus of interest, using 1.5 μL GeneJuice transfection reagent (Sigma-Aldrich). After a 24 hour incubation, plates were frozen and thawed, cells and supernatant were collected and transferred into 1.5 mL Eppendorf tubes for storage. CARVE-mediated selection was carried out similarly, with 10 μL of crude viral lysate was used to infect 70-80% confluent U2OS-Cas9 cells in 24-well plates for 2 hours. Media was changed to OptiMEM and 1.3 μL of 3 μM sgRNA stock was transfected using 1.5 μL GeneJuice. After a 24 hour incubation, plates were frozen and thawed, cells and supernatant were collected and transferred into 1.5 mL Eppendorf tubes for storage.

For assessment of antiviral effects of CRISPR/Cas9 using B8R targeted sgRNAs, U2OS-Cas9 cells in 12 wells were infected as described above, with a lower MOI (0.01), and infections were allowed to proceed for 72 hours. 72 hours post-infection, supernatants were harvested, and viral titers were then measured by standard plaque assays using crystal violet staining.

### Plaque scoring for quantification of recombinant VACV

Confluent U2OS cells were infected with lysates derived from CRISPR-assisted recombination at several dilutions to ensure wells with distinct viral plaques. Following a 2 hour infection in DMEM (Gibco), media was removed and replaced with overlay medium (DMEM supplemented with 10% FBS and 1.5% carboxymethylcellulose). Following 24 hour incubation, at least 60 plaques were counted plaques positive for fluorophore expression were graded based on detection on significant fluorescence over background (judged by mock-infected cells).

### Generation of B8R-targeted disA encoding donor vector

The homologous targeting sequences (left flank and right flank) were obtained from the VACV genomic reference sequence (Copenhagen vacc_M35027). The B8R targeting cassette was designed *in silico* to include: left homology flank (-1011 to -11 bases upstream of B8R start codon); pLate promoter-XhoI-hIL12p35-NotI; p7.5 promoter-eGFP; right homology flank (+670 to +1670 downstream of B8R start codon). The *in silico* generated B8R targeting cassette was synthesized by GenScript (Piscataway, NJ, USA) using the pUC57 Kan simple cloning vector (pUC57-B8R-hIL12p35-eGFP). PCR amplified XhoI-Disa-NotI DNA amplicon was ligated into XhoI/NotI digested pUC57-B8R-hIL12p35 backbone to create pUC57-B8R-Disa-eGFP (see **Supplementary Table 1** for sequence of primers). STINGPOX was generated using this vector and clonality of the recombinant virus was confirmed *via* DNA sequencing.

### Immunoblot analysis of primary mouse dendritic cells

Wild-type C57BL/6 and cGAS-deficient mice (Stock no: 026554) were purchased from Charles River Laboratories and The Jackson Laboratory, respectively. All animal experiments were approved by the Institutional Animal Research Ethics Board and concurred with the guidelines established by the Canadian Council on Animal Care. Myeloid DCs were prepared as previously described (30). Briefly, bone marrows were flushed from mouse femurs and tibias. The dissociated bone marrow cells were resuspended in complete RPMI 1640 in the presence of 55 μM of 2-mercaptoethanol (Gibco) and recombinant murine GM-CSF (40 ng/ml; PeproTech). On days 3 and 6, the cultures were provided with new media and fresh GM-CSF. On Day 7, DCs were harvested for the infection with various recombinant viruses as specified. For Western blot analysis, the treated cells were lysed in radioimmunoprecipitation buffer (RIPA) (10 mM phosphate pH 7.4, 137 mM NaCl, 1% NP-40, 0.5% sodium deoxycholate and 0.1% SDS) supplemented with protease inhibitor cocktail (Roche) and phosphatase inhibitor cocktail II and III (Sigma). Total cellular extracts were resolved on 7.5% SDS-PAGE and transferred to nitrocellulose membrane (Santa Cruz). Blots were blocked in Odyssey blocking buffer (Li-cor) and detection was performed with respective primary antibodies, and bands were visualized with infrared dye-conjugated secondary antibodies using Odyssey scanner (Li-cor).

The primary antibodies used for Western blotting were from the following sources: Abcam: IFIT1 (ab11821); Cell Signaling Technology: phospho-TBK1 (S172, #5483), TBK1 (3504), phospho-IRF3 (S396, #4947), IRF3 (#4302), phospho-STING (S365, #72971), STING (#3337), FLAG (#14793), cGAS (#31659); Sigma: β-actin antibody (A5441).

### RNA-sequencing analysis of primary human macrophages

Primary human macrophages (Stem Cell Technologies, Canada) were thawed and cultured in ImmunoCult™-SF Macrophage Medium (Stem Cell Technologies Canada) with recombinant M-CSF incubated at 37°C with 5% CO_2_. Macrophage M1 differentiation was achieved four days post-thaw by treatment with lipopolysaccharide and IFNγ. Differentiated macrophages were infected (MOI = 3) for 18h with either OncoVACV or STINGPOX. Cells were lysed with RLT buffer supplemented with 2-mercaptoethanol (1% v/v). Lysates were processed through a QIAshredder column (Qiagen), and then RNA was isolated using the RNeasy Mini kit (Qiagen) following manufacturer’s protocol. Approximately 1 µg of RNA was sent to FASTERIS for Illumina HiSeq 4000 paired-end 150 base pair (bp) read sequencing, with over 38 million sequencing reads.

Base-scaling was performed using HiSeq Control Software HD3.4.0.38, RTA 2.7.7 and bcl2fastq2.17 v2.17.1.14. The resulting fastq files were trimmed for adapter removal using Trimmomatic v0.33 (31). Trimmed reads were then mapped to the latest Ensembl human genome (v95) using HISAT2 (32). Relative mRNA levels of annotated protein coding genes were then estimated as transcripts per million (TPM) using StringTie (33). To calculate fold change between uninfected and infected samples, a pseudoTPM value of 1 was added to all genes in all conditions. Data was processed using the R statistical software (34) and displayed as a heatmap using the GraphPad Prism 9.0. RNA-seq data have been deposited to the Gene Expression Omnibus under the accession number: GSE214751.

Gene ontology analysis was performed using the ToppGene suite (35). For ToppGene biological process enrichment analysis, a gene list comprising of all genes activated over 10-fold in STINGPOX infected primary human macrophages relative to OncoVACV infected cells was used as an input. The *p* values shown were adjusted with Bonferroni correction. Heatmap visualization of RNA-seq data was done by further filtering this list of genes for those associated with the following biological processes: cytokine-mediated signaling pathway, response to cytokine, cellular response to cytokine stimulus, and innate immune response. All data shown is log2 transformed expression fold changes relative to mock conditions.

### Quantitative reverse transcriptase polymerase chain reaction

Aurum Total RNA Mini Kit (Bio-Rad) was used to perform RNA isolations, as per manufacturer’s protocol. Superscript II (Life Technologies) or iScript cDNA synthesis kit (Bio-Rad) were utilized for cDNA synthesis. Quantitative Polymerase Chain Reaction (qPCR) was performed on an Applied Biosystems Fast 7500 using SYBR Green Supermix (Bio-Rad). Primers used for qPCR are listed in **Supplementary Table 1**. A 20 μL reaction was assembled according to the manufacturer’s protocol. The 2^− ΔΔCt^ method was used for analysis and mean fold changes in expression relative to mock or control infected samples are shown.

### Cyclic di-AMP ELISA

HeLa cells were infected with OncoVACV or STINGPOX (MOI = 0.1) and 48 hours post-infection cells and supernatants were harvested. Cells were lysed in mammalian protein extraction reagent (M-PER; Thermo Scientific) and boiled at 95°C for 5 minutes. Cyclic di-AMP levels were measured in the cell lysate and supernatant *via* the cyclic di-AMP ELISA kit (Cayman Chemicals), as per manufacturer’s protocol.

### IFN-β ELISA

THP1-Dual cells were infected at MOI of 0.1 or 3 with OncoVACV or STINGPOX and, 24 hours post-infection, supernatants were harvested. IFN-β levels in supernatants were measured using the Human IFN-beta Quantikine ELISA kit (R&D Systems), as per the manufacturer’s protocol.

### Immunoblot analysis of cancer cell lines

Cells were lysed in 1X SDS lysis buffer (50 mM Tris-HCl, pH 6.9, 2% SDS and 10% glycerol) or in radioimmunoprecipitation assay buffer (RIPA buffer, ThermoFisher Scientific; 89901). Both lysis buffers were supplemented with 1x protease/phosphatase inhibitors (PPI 100x, 5872S; Cell Signaling Technology; 5872S). Protein concentrations were determined by bicinchoninic acid assay (BCA kit, 23227; ThermoFisher Scientific) or DC Protein Assay (Bio-Rad) according to the manufacturer’s protocol. Prior to loading, samples were mixed with 1x NuPage LDS sample buffer (LDS 4x, ThermoFisher Scientific; NP0007) or a combination of DTT and bromophenol blue. 30–60 µg was loaded per well into a pre-cast SDS/PAGE gel (Bio-Rad or Thermo-Scientific). Resolved proteins were transferred to nitrocellulose membranes using Mini-Protean Tetra System Trans-Blot Cell (Bio-Rad) or Trans-Blot Turbo System (Bio-Rad). Blots were blocked with Tris-buffered saline with tween (TBST) with 3% milk, washed with TBST, and subsequently probed with either a mouse anti-spCas9 (1:1000 dilution; Diagenode, C15200203), rabbit anti-GAPDH (1:5000; Cell Signaling Technology; 2118S), mouse anti-FLAG (1:1000; Sigma-Aldrich; F1804) or rabbit anti-PD-L1 (1:1000; Abcam; ab213480). Where indicated, total protein was assessed *via* Ponceau Stain (Sigma-Aldrich; P7170-1L). After overnight incubation at 4°C, blots were washed with TBST and probed with horseradish peroxidase-conjugated secondary antibodies (1:3000-1:5000; Cell Signaling Technology). Blots were visualized using Bio-Rad Clarity ECL.

### Screening of bacterial cyclases in mammalian cells

The FLAG-tagged human codon optimized sequences of seven different cyclases were cloned by GenScript into a CMV-driven mammalian expression plasmid (pCDNA3.1(+) plasmids). The protein coding reference sequences are listed in **Supplementary Table 2**. Expression in HEK 293T cells was confirmed by immunoblot (data not shown). pCDNA3.1(+) plasmids expressing various WT bacterial cyclases were transfected into 293T cells at 70% confluency in 60 mm plates using Roche X-tremeGENE™ HP DNA transfection Reagent (Roche), as per manufacturer’s protocol. 24 hours post-transfection, lysates containing the cyclic dinucleotides synthesized by the cyclases were prepared. Briefly, the transfected cells were transferred to 15 mL Falcon tubes, and pelleted at 500 RCF at for 5 min. The cell pellets were resuspended in a hypotonic solution (10 mM Tris-HCL, pH 7.4, 10 mM KCL, 1.5 mM MgCl_2_) with Dounce homogenizer. The homogenates were centrifuged at 21,000 RCF for 5 min to remove large cellular particles and the supernatants were heated at 95°C for 5 min, then incubated on ice for 5 min; then clarified at 21,000 RCF at 4°C for 10 min to remove denatured proteins. The resulting supernatants were diluted and used to treat THP1-Blue ISG reporter cells to measure induction of IFN signaling.

### THP-ISG-blue, 293-dual hSTING-R232, and THP1-dual cell reporter assays

Assays using these reporter cell lines were performed as per manufacturer’s protocol. Where noted, c-di-AMP and c-di-GMP (5 µg/mL; InvivoGen; tlrlnacda and tlrlnacdg) or Lipofectamine 2000 transfected immunostimulatory DNA (ISD; InvivoGen; tlrl-isdn) were used as positive controls in assays. For ISD transfection, the Lipofectamine 2000 complexed ISD was added at a concentration of 4 µg/ml for 24 h. For SEAP reporter assays, the color densities were determined with a microplate reader at OD 630 nm. Data representative of 3 independent experiments. For luciferase assays, measurements were made in solid white-bottom plates using a BioTek Microplate Reader.

### Human tumor explant analysis

For growth in patient tumor cores, three 2 mm x 2 mm cores were randomly distributed in 24-wells with 500 μL of DMEM + 10% FBS media. Infections with OncoVACV and STINGPOX were done at 1E4 PFU per well. Two hours post-inoculation, cores were washed with PBS and media was replaced. 72 h post-infection, explants were collected into Eppendorf tubes and then the entire tube was flash frozen in liquid nitrogen. Using dry ice cooled sterilized mortar and pestle, the tumor sample was ground into a fine powder. Then tumor sample was then placed in collection tubes (2.0 mL colorless Eppendorf safe-lock tubes) that were pre-cooled in dry ice. Following that, 350ul RLT (Aurum Total RNA Mini Kit) was added with 1% β-mercaptoethanol (Sigma-Aldrich) to each sample with 5mm stainless Steel Bead (QIAGEN) and placed in the TissueLyserII (QIAGEN - 2 runs: 30Mhz, 2min). Tubes were then spun down at 21,000 RCF and the supernatants were transferred into QIAshredder (Qiagen). RNA extraction was then performed using Aurum Total RNA Mini Kit (Aurum Total RNA Mini Kit) following the manufacturer’s protocol.

### *In vivo* mouse experiments

MC38 cells were washed twice with PBS and filtered (0.45 µm) and then 5E5 cells in 100 µL of a 1:1 solution of PBS and Matrigel (Corning; 356231) were injected subcutaneously in the right flank of 7-week old immunocompetent C57BL/6 mice (Jackson) with Matrigel (Corning). Mice were randomized following tumor implantation. Intratumoral injections of virus (3x 1E7 PFU in 100 µL PBS) were performed on days 6, 8, and 10 post-tumor implantation. As a control, other groups received 100 µL PBS. Intraperitoneal injections of anti-PD-1 (clone RMP1-14; approx. 100 µg in 100 µL; BioXCell; BE0146) or rat IgG2a isotype control (clone 2A3; 5 mg/kg in 100 µL; BioXCell; BE0089) were also performed on days 10, 14, 17, 21, 24, and 31 post-tumor implantation. Tumor sizes were recorded 2-3 times a week. Each experimental group contained 10 mice. Statistical analyses were conducted in GraphPad Prism (CA, USA).

### Statistical analyses

One-way or two-way ANOVA were performed where appropriate, followed by Tukey’s tests for pairwise comparisons. For survival curve analyses, Log-rank (Mantel-Cox) Test was performed on GraphPad Prism 9.0.

## Results

### Development of CRISPR-assisted recombinant VACV engineering system

The objective of the current study was to generate an adaptable, modular system which would enable the rapid rescue of various VACV strains with transgene incorporation and/or attenuation at multiple viral genome loci without the need for extensive cloning and minimal construct size. To this end, we designed a simple CRISPR/Cas9-based approach for transgene insertion into the VACV genome requiring only a single guide RNA (sgRNA) and a compatible homologous recombination template (HRT) containing a sequence of interest. sgRNAs were designed with the highest “on-target” score targeting residual wildtype alleles (28). HRTs have been designed with 500 bp homology arms that flank the sgRNA target sequence, resulting in the removal of the target and insertion of a fluorescent reporter. HRT and sgRNA transfection was delayed 2 hours following infection to allow for the initiation of viral DNA replication, creating an environment more conducive to homology directed repair (HDR).

Similar to their utility in large genomic screens, a stable cell line expressing Cas9 provides a simple, scalable and affordable method of Cas9 delivery (36). This design should enable the use of a single cell line for any genomic insertion site, with the use of an sgRNA targeting the loci of interest and a compatible HRT. U2OS cells stably overexpressing Cas9 lacking an added NLS (in line with cytoplasmic localization of VACV replication complexes) were generated transduction with lentivirus produced using pCMV-TO Puro-Cas9. In order to ensure robust expression and cleavage activity, a single clonal population with the highest Cas9 expression was expanded (37). (**Supplementary Figure 1**).

To initially validate the capacity of our system to accelerate recombinant VACV rescue, sgRNA and HRT sequences were designed to facilitate the insertion of a fluorescent marker into the B8R locus in Copenhagen strain of VACV. The B8R gene encodes a soluble IFN-γ scavenger, representing an important VACV immune modulator (38). An HRT with 500 bp homology arms was designed to insert EGFP coding sequence under control of the B8R promoter, while eliminating the sgRNA target sequence. Transfection of the sgRNA in the U2OS-Cas9 cells following infection with Copenhagen reduced viral titers, consistent with Cas9-mediated cleavage of the B8R locus (**Supplementary Figure 2**).

**Figure 1:**
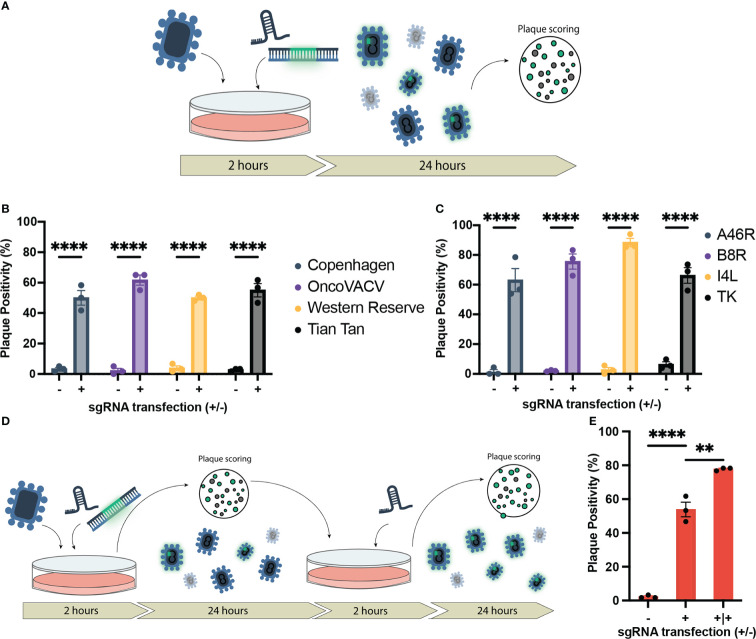
Establishment of CRISPR/Cas9-assisted recombinant VACV engineering (CARVE). **(A)** Schematic of basic recombination protocol. U2OS-Cas9 cells are infected with VACV progenitor strain and subsequently transfected with the homologous recombination template and corresponding synthetic guide RNA for the loci of interest. Bulk virus populations are then collected after 24 hours and used to infect monolayers to score for fluorescence. **(B)** Proportion of plaques positive for EGFP inserted at the B8R loci following recombination in various Vaccinia virus strains. U2OS-Cas9 cells were infected with model VACV strains (multiplicity of infection (MOI) = 0.1) and subsequently transfected with the B8R homologous recombination template (HRT) with and without the B8R-targeting single-guide RNA (sgRNA). **(C)** Proportion of plaques positive for fluorescent marker following recombination of VACV Copenhagen at various loci (B8R, A46R, J2R, and I4L). Cells were infected at MOI = 0.1 and transfected with the HRT targeting the domain of interest with or without the corresponding guide (n = 3). **(D)** Outline of CRISPR-Cas9 selection following recombination to enhance recombinant rescue rate. Progeny virus from first step **(A)** is used to inoculate U2OS-Cas9 cells, which are then transfected with the sgRNA to facilitate selection. **(E)** Enhancement in proportion of plaques positive for mCherry inserted in the J2R locus following first-round recombination and a single round of selection (n = 3). Data are shown as mean ± SEM (p**<0.1, p****<0.001).

To measure the impact of the HRT and sgRNA co-transfection on recombination efficiency, bulk output viral population was collected 24 hours following infection/transfection and then scored for fluorescence (reporter for recombinant viral clones) using a plaque assay on wild-type U2OS cells (outlined in **Figure 1A**). As a control, we also performed the infection/transfection protocol in the absence of the corresponding sgRNA to mimic the traditional infection/transfection protocol. When the B8R-targeted sgRNA and HRT were co-transfected following infection, the proportion of EGFP positive plaques was significantly increased from 3.3% to 50% (**Figure 1B**). This increase in recombination rate was also observed when targeting three other VACV strains of clinical significance; Western Reserve, Tian Tan, and OncoVACV (a novel attenuated oncoselective VACV vector with major deletions of several immunomodulatory virulence factors) (**Figure 1C**) (25). Cas9 expression in this U2OS-Cas9 stable cell line is both robust and long-lasting with maintenance antibiotic selection and improved recombination efficiency (**Supplementary Figure 3**).

To examine the versatility of this approach, HRTs and sgRNAs were designed for three additional VACV loci. These loci included attenuating knockouts of: J2R, encoding a thymidine kinase; I4L, a ribonucleotide reductase subunit; and A46R, encoding a toll-like receptor mimic (39–41). Disruption of J2R and I4L attenuate the virus in healthy cells without abundant cytoplasmic deoxynucleotide triphosphate pools, while A46R, like B8R, plays an immune modulatory role. These attenuating mutations can improve the safety of VACV, while also increasing the tumor selectivity of the VACV backbone as an oncolytic, since the attenuations have less impact on virus growth in cancer cells compared to normal cells (42–44).

In all loci tested, there was a marked improvement in the first-round recombination frequency, only 24 hours after infection/transfection (up to 66-88%) (**Figure 1C**). With the increase in first-round recombination rates, we explored if additional selection steps could be mediated by the sgRNA activity. Bulk viral output from the first-round of recombination was used to infect U2OS-Cas9 and, two hours post-infection, the corresponding sgRNA was transfected again to mediate selection against the parental strain (**Figure 1D**). One additional round of selection following first-round recombination facilitated an increase in mean plaque positivity for J2R insertion in Copenhagen from 53.9% in the first-round to 77.8% (**Figure 1E**). Collectively, the data demonstrates that our methodology accelerates generation of recombinant VACV vectors. We termed this system CRISPR-assisted recombinant VACV engineering (or CARVE).

### CARVE system enables rapid-multi arming of VACV-based vectors

**Figure 2:**
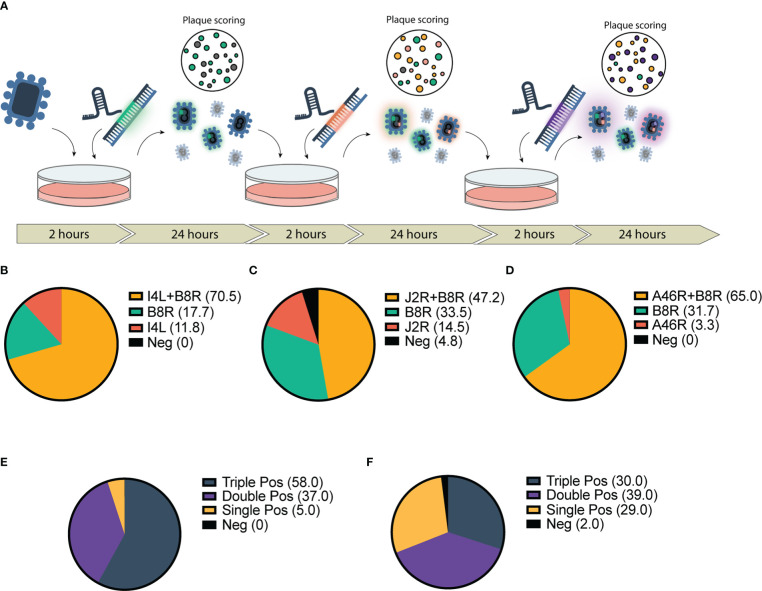
Application of CRISPR-mediated rapid rescue protocol to the accelerated rescue of multi-armed Vaccinia virus. **(A)** Timeline for rapid multi-arming of Vaccinia virus Copenhagen at multiple viral loci. Bulk virus is collected and used as inoculum for following infection/transfection and stored for scoring. **(B–F)** Scoring of bulk viral output following two-step infection transfection of Cop -B8R-I4L **(B)** Cop -B8R-J2R **(C)** and Cop -B8R-A46R **(D)** (n = 3). Three-step recombination resulting in Cop -B8R-I4L-A46R **(E)** and Cop -B8R-J2R-A46R **(F)** (n = 2). Loci are labelled in order of CARVE-mediated insertion of EGFP (B8R), mCherry (I4L and J2R), and iRFP720 (A46R).

Given the marked increase in first-round recombination efficiency, we predicted that the CARVE system could facilitate rapid multi-arming of VACV. We took the bulk output populations from a single round of recombination at our various loci and performed a second round of recombination targeting a second locus (**Figure 2A**). The result was a large population of double positive plaques populations (47.2%-70.5% of the population) (**Figures 2B–D**). The bulk populations of this second-round were then subjected to a third and triple recombinant viruses were easily identified within 72 hours of the initiation of the experiment (30-58% of the population) (**Figures 2E, F** and **Supplementary Figure 4**). When combined with two rounds of standard plaque picking, a pure triple insert virus clonal population was achieved in as few as 7 days, clonality was confirmed by PCR (**Supplementary Figure 5**).

### CARVE-mediated rapid generation of recombinant VACV vector expressing immunogenic payload

Viral vectors are employed in vaccines due to the induction of immune responses without the need for an adjuvant. In the case of poxvirus vectors, the immunomodulators encoded by poxvirus vectors likely dampen their immunogenicity (45). Using THP1 cells bearing a SEAP reporter whose expression was driven by interferon response factor (IRF) activity (THP1-Blue ISG cells), we confirmed that the deletion of a subset of these immunomodulators to generate attenuated OncoVACV yields a virus whose infection induces higher levels of IFN signaling activity than its parental Copenhagen strain (**Supplementary Figure 6**) (25). The OncoVACV genome harbors deletions of viral factors known to inhibit IFN signaling and production. However, relative to the highly attenuated replication deficient modified vaccinia Ankara (MVA), OncoVACV infection did not drive as robust of an IFN signaling response. MVA is known to induce IFN signaling through the STING pathway (46); however, its potential for transgene delivery is limited due to its replication deficiency in mammalian cells. We hypothesized that encoding a STING agonist in OncoVACV backbone could generate a highly immunogenic viral vector that drives STING signaling in the tumor microenvironment while maintaining its replicative capacity. The oncoselective properties of OncoVACV would provide a tumor-specific vector for delivery of STING agonists, limiting the adverse events associated with systemic application of STING agonists. Both bacteria and mammals utilize cyclic dinucleotides as secondary messengers, so we scanned for candidate cyclases for incorporation into our vector. While we considered the incorporation of the metazoan dinucleotide cyclase, 2’-3’-cyclic GMP-AMP (2’-3’-cGAMP) synthase (cGAS), recent studies have revealed that VACV encodes 2’-3’-cGAMP-specific nucleases which would dampen the desired immunogenic effects from a virally encoded cGAS (47). Several bacteria encode dinucleotide cyclases that catalyze the synthesis of cyclic dinucleotides (CDNs), such as cyclic 3’-3’-GMP-AMP (3’-3’-cGAMP), cyclic di-AMP (c-di-AMP), and cyclic di-GMP (c-di-GMP). All of these bacterial secondary messengers also act as STING ligands in mammalian cells, agonizing the pathway, and have been considered for use as vaccine adjuvants. Therefore, we encoded a bacterial cyclase in OncoVACV to create an oncolytic virus capable of producing STING agonist *in situ* during infection.

**Figure 3:**
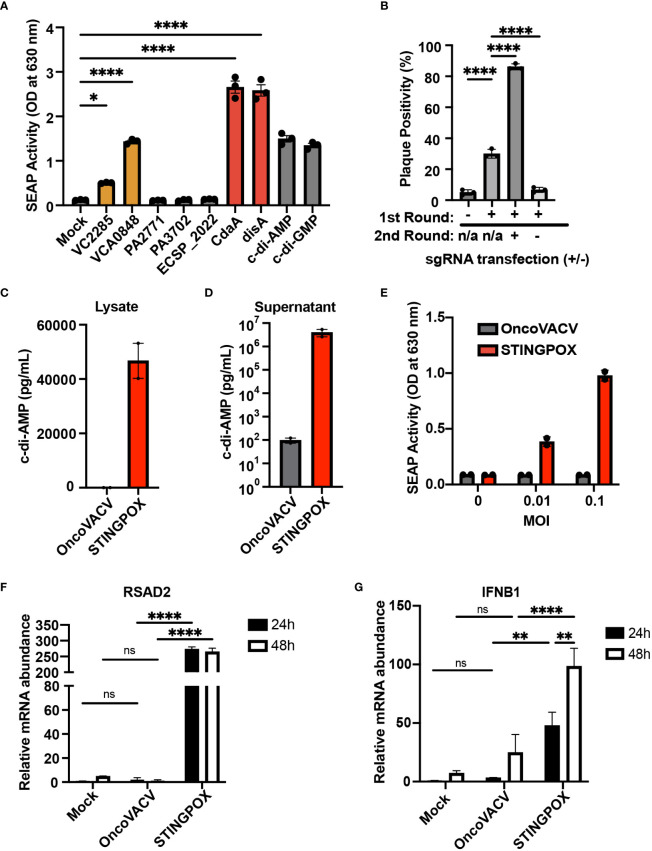
CARVE-mediated generation of novel immunotherapeutic STINGPOX. **(A)** Mammalian expression plasmid screen of bacterial cyclases. HEK293T were transfected with expression plasmids for noted cyclase and then lysed 24 hours post-transfection. Diluted clarified lysates were then used to treat THP- Blue ISG cells, and SEAP reporter assay was performed to measure relative effect on IFN signaling (n=3). **(B)** CARVE-mediated rescue of OncoVACV +MtbDisA inserted at the B8R locus. Virus underwent traditional CARVE protocol with transfection of HRT encoding DisA (1st round) with or without sgB8R, virus produced with sgB8R in this step underwent a second round of selection where sgB8R was transfected alone, following infection. Resulting virus was used to infect confluent U2OS and plaques were scored for EGFP fluorescence (n = 3). **(C, D)** Cyclic di-AMP ELISA was used to measure cyclic di-AMP levels in HeLa cell **(C)** lysates and **(D)** supernatants - 48 hours post-infection with STINGPOX or OncoVACV (n = 3). **(E)** 293-Dual hSTING-R232 cells SEAP reporter assay was performed to measure effects of infection with STINGPOX or OncoVACV on IFN signaling (48 hours post-infection; n = 3). **(F, G)** qRT-PCR analyses of relative **(F)** IFNB1 and **(G)** RSAD2 mRNA levels in HT29 cells, 48 hours-post infection with STINGPOX or OncoVACV (MOI = 0.1; n≥2). Data are shown as mean ± SEM (p* < 0.05, p** < 0.01, p**** < 0.001), not significant (ns).

We performed an overexpression screen with bacterial cyclases to identify bacterial CDN synthesizing enzymes resulting in potent activation of the STING signaling cascade in mammalian cells. Dinucleotide cyclases were selected from pathogenic bacteria: *Vibrio cholerae* (VCA0848 and VC2285)*, Pseudomonas aeruginosa* (PA2771 and PA3702)*, Escherichia coli* (ECSP_2022)*, Listeria monocytogenes* (cdaA), and *Mycobacterium tuberculosis* (disA) (48–53). These bacteria were selected due to their viability at 37°C to increase the probability their dinucleotide cyclase activity would remain functional in mammalian cells. We screened the ability of these cyclases to induce STING/IRF signaling using THP1-Blue ISG cells. THP1-Blue ISG cells express functional STING, and, upon stimulation with a STING ligand, such as cyclic di-AMP, activate IRF signaling and IRF-driven expression of a SEAP reporter. The THP1 reporter cells were treated with clarified lysates from 293T cells transfected with mammalian expression plasmids for human codon optimized sequences of each cyclase. Among the seven cyclases screened, four cyclases were identified as driving STING/IRF signaling in THP1-Blue ISG cells: two diguanylate cyclases (VCA0848 and VCA2285) and two diadenylate cyclases (cdaA and disA) (**Figure 3A**). The cyclases cdaA and disA, in particular, catalyze c-di-AMP production and demonstrated robust capacity to drive STING signaling in the reporter cells. As disA was previously shown to increase the immunogenicity of a tuberculosis vaccine in pre-clinical studies (54), we selected disA for incorporation as a transgene in OncoVACV for our pilot studies.

While encoding an enzyme producing a STING agonist in a VACV-based vector would increase its immunogenicity, it could in turn hinder viral replication and subsequent purification in any cell line possessing active STING signaling (e.g. U2OS). Therefore, we applied the CARVE approach to accelerate purification of OncoVACV expressing disA in the B8R locus in U2OS cells. In the presence of a guide RNA targeting B8R (sgB8R), we were able to enrich the OncoVACV to over 86% purity, consistent with our initial experiments validating the CARVE approach (**Figure 1B**). In the absence of sgB8R, OncoVACV has greater fitness than OncoVACV-disA, making it challenging to isolate recombinants by the traditional methods (**Figure 3B**). These results demonstrated that the CARVE approach accelerates the generation of recombinant poxvirus vectors expressing immunogenic payloads, including those which may result in a reduction in viral fitness. As OncoVACV-disA is predicted to drive STING signaling, we renamed the immunotherapeutic candidate vector STINGPOX.

### STINGPOX infection enhances IFN signaling *in vitro*

Next, we characterized STINGPOX’s effects on IFN signaling. STINGPOX infection should result in expression of disA and production of c-di-AMP, a STING agonist driving downstream IFN signaling. By performing a c-di-AMP ELISA, we validated that infection of HeLa cells with STINGPOX resulted in c-di-AMP production intracellularly (**Figure 3C**). Further ELISA analysis also demonstrated release of c-di-AMP into the supernatant - likely due to virus-induced lysis of the HeLa cells (**Figure 3D**). We examined the ability of STINGPOX to induce IFN signaling in infected cells using 293-Dual hSTING-R232 cells, which harbor a fully functional STING/TBK1/IRF3 signaling and express a secreted embryonic alkaline phosphatase (SEAP) reporter under the control of an IFN regulatory factor (IRF)-inducible promoter (**Figure 3E**). Activation of STING signaling in 293-Dual hSTING-R232 cells will result in downstream IRF signaling and increased expression of the SEAP reporter. STINGPOX activated IRF signaling in a virus concentration-dependent fashion, whereas infection with OncoVACV did not induce SEAP activity. qRT-PCR analyses of infected HT29 cell lines, a human colorectal cancer cell line possessing deficient, but still active signaling, demonstrated that STINGPOX activated IFNB1 (a type I IFN) and RSAD1 (a downstream interferon stimulated gene; ISG) expression to significantly higher levels than the parental OncoVACV (**Figures 3F, G** **)**. These results demonstrate that STINGPOX drives IFN signaling more potently than OncoVACV.

**Figure 4:**
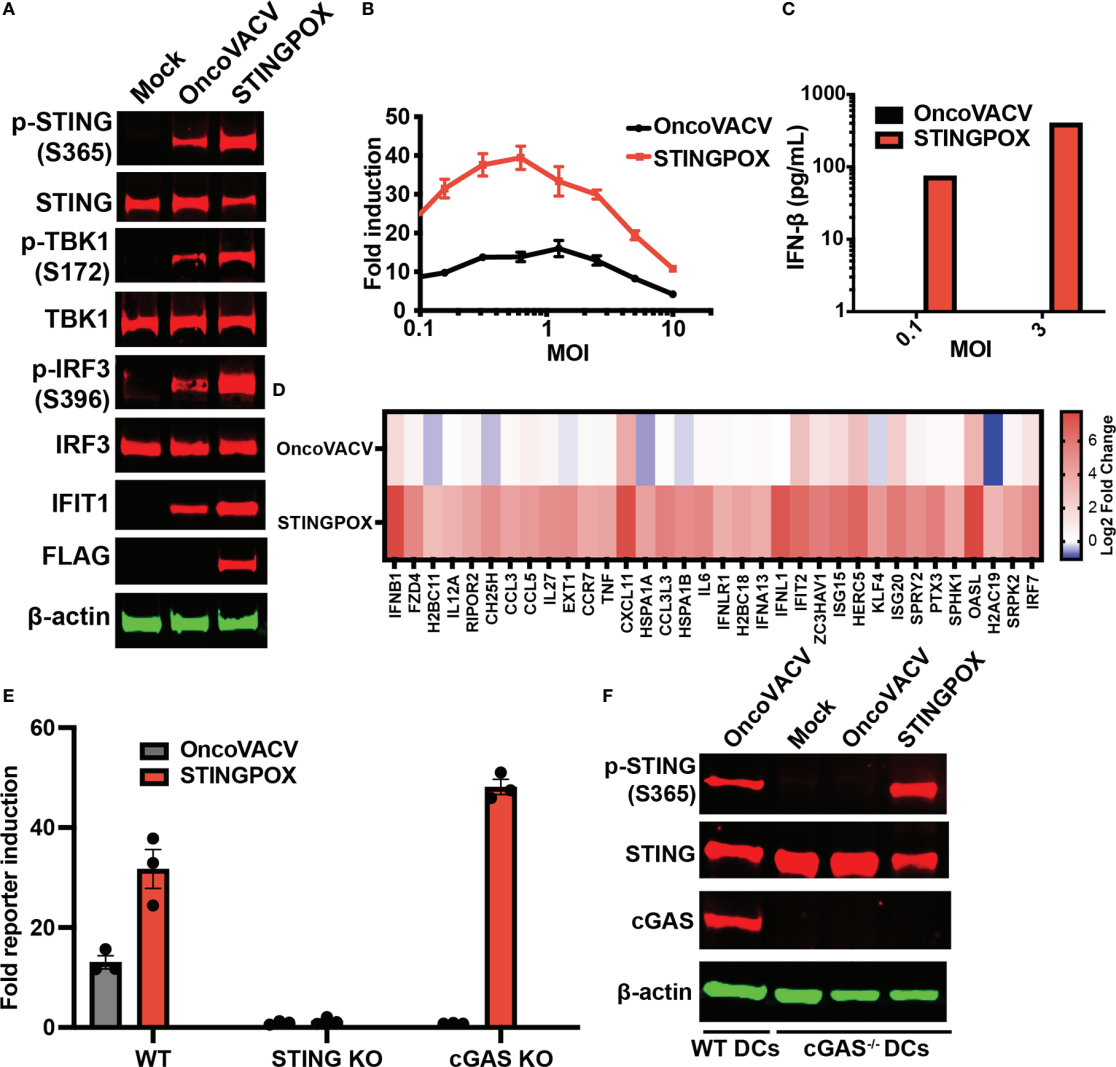
STINGPOX induces IFN signaling in a STING-dependent manner. **(A)** Primary murine BMDCs were infected with STINGPOX or OncoVACV for 24 h. Whole cell lysates were analyzed by WB for phospho-STING (p-STING S365), STING, phospho-TBK1 (p-TBK1 S172), TBK1, phospho-IRF3 (p-IRF3 S396), IRF3, IFIT1, cGAS and FLAG (for disA expression). β-actin levels were used as loading control. Data are representative of three independent experiments. **(B)** THP1-Dual cells were infected with STINGPOX or OncoVACV (at a range of MOIs) and relative luciferase activity was assessed as a measure of IFN signaling (n = 3). **(C)** Supernatants IFN-β levels in THP1-Dual infected with STINGPOX or OncoVACV (MOI of 0.3 or 1) for 24 h were measured by ELISA. **(D)** Heatmap of relative fold change in expression (to mock) in primary human macrophages infected with OncoVACV or STINGPOX (MOI = 3) for 18 hours, and analyzed by RNA-seq. Log2 transformed data is shown for genes associated Jak/STAT or IFN αβ signaling pathways that were activated at least 4-fold in STINGPOX infected cells relative to OncoVACV infection conditions. **(E)** IFN signaling measured as described in **(B)** using WT, cGAS KO, and STING KO THP1-Dual cells. **(F)** Immunoblot analyses of primary murine WT and cGAS^-/-^ BMDCs infected with STINGPOX or OncoVACV, as described in **(A)**. Data are shown as mean ± SEM.

Recent studies have demonstrated that activation of STING signaling in dendritic cells (DCs) is critical to the induction of anti-tumor immunity, including generation of tumor cell-specific CD8+ T cells (55), so we investigated the effects of STINGPOX on DCs. Immunoblot analyses revealed that STINGPOX infection of primary murine bone-marrow derived DCs (mBMDCs) resulted in increased phosphorylation of STING, TBK1, and IRF3 relative to OncoVACV (**Figure 4A**). Furthermore, increased expression of IFIT1, an ISG, was also observed. These results demonstrate that STINGPOX activates STING/IFN signaling in dendritic cells. Similarly, we also demonstrated that STINGPOX drives IRF signaling in THP1-Dual cells that stably express a luciferase reporter under the control of an ISG promoter (**Figures 4B, C** **)**. ELISA analysis of the supernatants of infected THP1-Dual cells for IFN-β secretion demonstrated, as expected, that STINGPOX drives IFN production (**Figure 4C**). To validate STINGPOX’s ability to drive IFN production and signaling in primary human immune cells, we performed RNA sequencing analysis on human macrophages infected with STINGPOX or OncoVACV. Pathway enrichment analysis on the list of genes expressed 10-fold higher in STINGPOX infected cells relative to OncoVACV infected cells revealed that STINGPOX infection resulted in greater expression of genes associated with cytokine and innate immune signaling (**Supplementary Table 3** **;** **Figure 4D**), in line with our previous results (**Figure 3**).

Lastly, we examined the mechanism by which STINGPOX drives the IFN signaling axis. As STINGPOX-mediated expression of *disA* drives production of c-di-AMP, a known ligand of STING, we postulated that STING would be critical to the virus’s ability to drive IFN signaling. Consistent with this, STING knockout in THP1-Dual cells eliminated STINGPOX’s ability to enhance IRF-driven luciferase activity (**Figure 4E**). On the other hand, knocking out cGAS, an endogenous mammalian cyclase upstream of STING, did not impair STINGPOX infection’s effects on IFN signaling. Independently, we validated this observation using primary cGAS^-/-^ mBMDCs, where STINGPOX’s enhancement of STING activation was not impaired (**Figure 4F**). Collectively, our results suggest that STINGPOX drives IFN signaling in a STING-dependent, cGAS-independent fashion.

### Anti-tumor effects of STINGPOX *in vivo*

**Figure 5:**
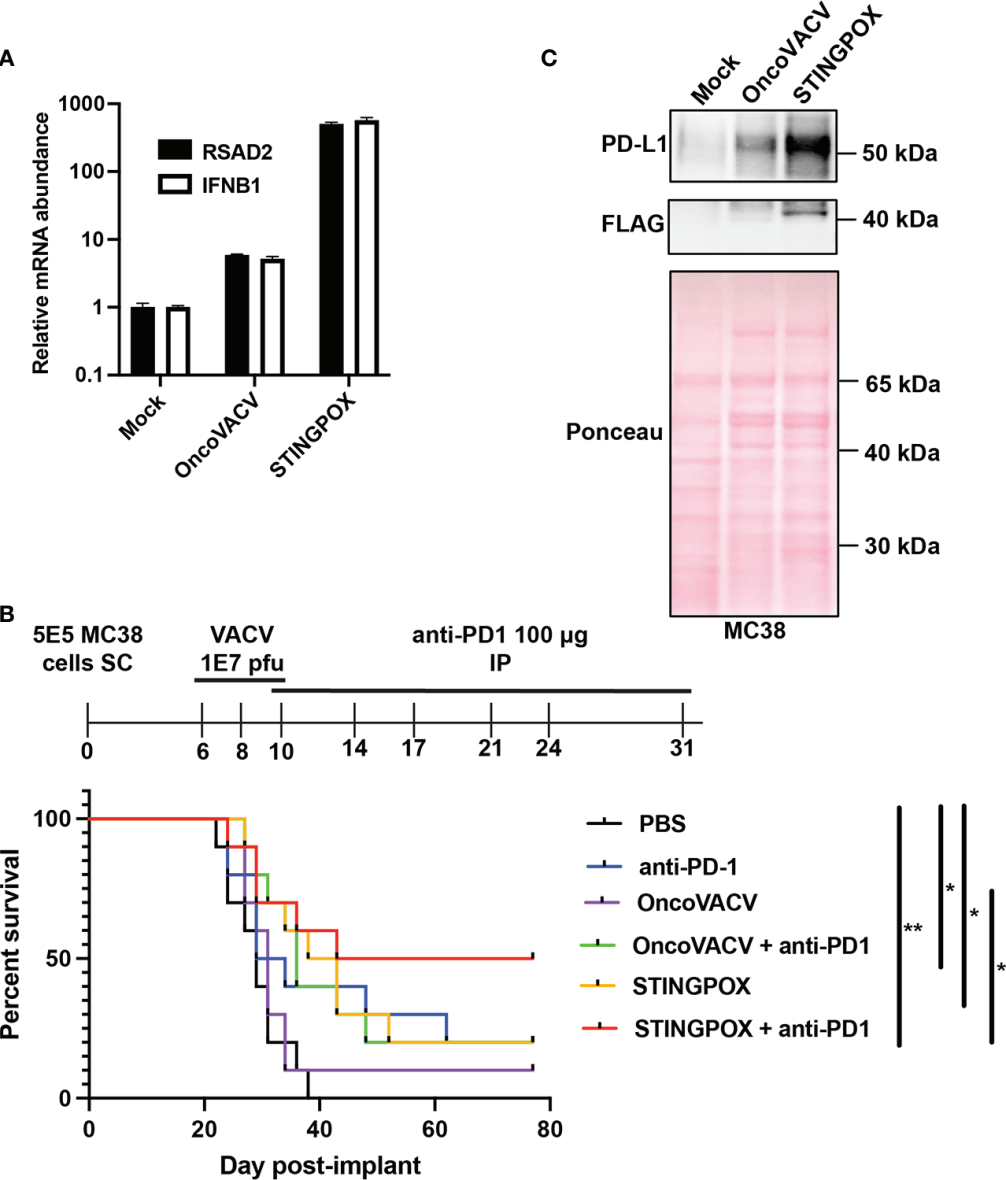
STINGPOX induces anti-tumor effects *in vivo*. **(A)** Ovarian tumor explants were infected with STINGPOX or OncoVACV (1E4 PFU per tumor core), and qRT-PCR analysis was performed 72 hours post-infection to measure relative RSAD2 (n=3) and IFNB1 (n≥2) expression levels. **(B)** Mice bearing subcutaneous (SC) MC38 colorectal tumor, were treated with 3 doses intratumorally of PBS, OncoVACV, or STINGPOX intratumorally (IT) and anti-PD1 intraperitoneally (IP) as indicated in the timeline. Mouse survival plots are shown. (n=10 per group; *p<0.05; **p<0.005). **(C)** MC38 cells were infected *in vitro* with OncoVACV-GFP and STINGPOX (MOI = 1), and 48 hours post-infection, whole cell lysates were analyzed by WB for PD-L1 and disA (FLAG) expression. Ponceau staining of blot is shown for loading control. Data are shown as mean ± SEM.

We investigated STINGPOX’s therapeutic potential in clinically relevant models. STINGPOX-induced IFN signaling was measured in a primary ovarian tumor explant model (**Figure 5A**). qRT-PCR analyses revealed that STINGPOX drives RSAD2 and IFNB1 expression in the ovarian tumor explants, consistent with our *in vitro* observations in cancer cell lines (**Figures 3F, G**). We then proceeded to evaluate STINGPOX therapeutic activity in an *in vivo* murine syngeneic immunocompetent model. Previous work had demonstrated that oncolytic poxviruses work in synergy with anti-PD-1/PD-L1 therapy, and this was linked to the viruses’ ability to drive IFN signaling and downstream expression of PD-L1, an ISG (56). We predicted that STINGPOX’s ability to drive IFN signaling at a higher level than OncoVACV would result in greater synergy with checkpoint therapy. We compared OncoVACV and STINGPOX’s anti-tumor effects in combination with anti-PD1 *in vivo*, using C57B/L6 mice bearing subcutaneous MC38 colorectal tumors (**Figure 5B** **;** **Supplementary Figure 7**). Our results demonstrated that the greatest survival was observed in the mice treated with STINGPOX and anti-PD1. Whereas OncoVACV and anti-PD1 resulted in 20% survival, STINGPOX and anti-PD1 combination therapy increased survival rates to 50%. In parallel, we confirmed *in vitro* that STINGPOX infection activated PD-L1 expression relative to OncoVACV in MC38 cells (**Figure 5C**). We also observed a similar increase in PD-L1 expression in B16-OVA cells during STINGPOX infection **(** **Supplementary Figure 8**). These results are consistent with clinical studies proposing a correlation between response to checkpoint therapy and tumor PD-L1 expression levels (57). Collectively, our observations suggest that the increased IFN signaling driven by encoding a diadenylate cyclase in STINGPOX synergizes with therapeutic strategies disrupting the PD-1/PD-L1 axis.

## Discussion

Vaccinia virus represents a promising tool in the prevention of infectious diseases as well as the treatment of malignancy, especially as this virus has a large transgene coding capacity, potentially enabling the expression of multiple therapeutic transgenes (2). However, empirical or strategic assessment of the activity of multiple interacting transgenes encoded within a virus has been limited by the tools to facilitate the manipulation of its genome. While several different approaches have been developed and refined over the years [e.g. essential gene rescue, antibiotic selection, flow-cytometry based approaches (58–61)], none of these protocols have become common-practice in the field, as many come with drawbacks such as being labor-intensive, inaccessible due to the requirement for specialized instruments, dependent on the use of markers, or lacking the adaptability to facilitate extensive genome modification.

We developed a simple, rapid and affordable system for the generation of recombinant VACV strains. We demonstrate a marked increase in first-round recombination rates at various loci across the genome, and in various strains when compared to traditional homologous recombination approaches (**Figure 1**). This increase enabled the rescue of a recombinant VACV Copenhagen carrying three transgenes in as little as 72 hours at a rate well above what would be expected in a single insertion using the traditional approach (**Figure 2**). Furthermore, this system is compatible with any of the aforementioned approaches; therefore, combinations of CARVE with essential gene rescue, antibiotic selection, flow-cytometry based approaches, has the potential to further increase the ease and speed with which large numbers of recombinant viruses could be isolated. When compared to these other methods, the only additional consideration in the CARVE system is the design and synthesis of a compatible sgRNA to be combined with the HRT. The design and synthesis of an HRT, by oligonucleotide synthesis or traditional cloning, is required for all engineering strategies, therefore CARVE does not introduce additional burden at this stage.

Previous work has demonstrated the feasibility of using CRISPR-Cas9 to facilitate the rescue of novel VACV strains encoding foreign transgenes (15, 62). In these studies, improved rescue of recombinants was attributed to different mechanisms. For instance, the increase in recombination rate could rely on the induction of homology-directed repair (HDR) following cleavage at the target site, or alternatively counter selection of unedited sequences. These two potential mechanisms of action are linked and not mutually exclusive. It is well documented that recombination in VACV is closely tied to replication, therefore any cleavage which is meant to induce HDR would need to be closely tied to the start of replication (63). Similarly, selection which takes place soon after early replication would provide a strong selective advantage to the recombinant population and give the appearance of an increased frequency of recombination. A recent report from Gowripalan et al. suggests the dominant mechanism is in fact the selection of the recombinant viral population which lacks the guide target sequence (15). Our results agree that this selection clearly plays an important role (**Figure 1E**); however, we believe that in our approach increased HDR is also a contributory factor. The differences from earlier results are likely due to our unique approach. We believe the dramatic and rapid enhancement of multiple gene insertions seen in this study may result from the differences in the delivery method of the Cas9. The delayed introduction of the sgRNA, separately from Cas9, through transfection delays the formation of the functional RNP, allowing for the initiation of viral replication. This could create a more conducive environment for HDR to take place. A simple outline of the CARVE system can be seen **Supplementary Figure 9**.

A concern in application of CRISPR/Cas9-mediated genome editing is the potential for off-target effects. These effects can arise from the spurious cleavage activity of Cas9 resulting in double-strand breaks elsewhere in the target genome leading to non-homologous end-joining (NHEJ)-mediated repair (64). It has been reported that NHEJ is a rare event in the context of the cytoplasmic VACV replication given its reliance on the nuclear DNA ligase IV (65). Furthermore, VACV replication is likely characterized by somewhat frequent double-strand breaks, which is followed by homologous recombination (8). Indeed similar Cas9-mediated approaches to VACV rescue have found relatively rare instances of NHEJ-mediated escape (15). With proper guide design, the risk of off-target modifications can be minimized.

As a proof-of-concept of the CARVE system’s ability to accelerate recombinant poxvirus generation, we developed a STING agonizing VACV vector, which we termed STINGPOX. While previous studies have encoded bacterial dinucleotide cyclases to improve immunogenicity of bacteria as immunotherapies (54, 66), or replication-deficient adenoviruses (50, 67), to our knowledge this is the first study to examine the utility of these enzymes in replicating viral vectors. In this study, we develop STINGPOX, a recombinant VACV encoding the *M. tuberculosis* disA gene. The disA enzyme has previously been applied to increase the immunogenicity of bacille Calmette-Guérin (BCG) tuberculosis vaccine. In this study, we utilize it to enhance the immunogenicity of an oncolytic virus. We demonstrate in primary immune cells, patient explants, and cancer cell models (**Figures 3**, **4**, **5A, C**) that STINGPOX induces expression of type I IFNs and downstream gene products (including PD-L1) following production of c-di-AMP. The expression of these pro-inflammatory gene products and cyclic dinucleotides appears to “*heat up*” immunologically cold tumors providing survival benefit in an immune competent colorectal cancer model, especially in the presence of a PD-1 immune checkpoint inhibitor (**Figure 5B**). These pre-clinical studies support further evaluation of oncolytic poxviruses encoding bacterial cyclases for cancer immunotherapy.

**Figure 6:**
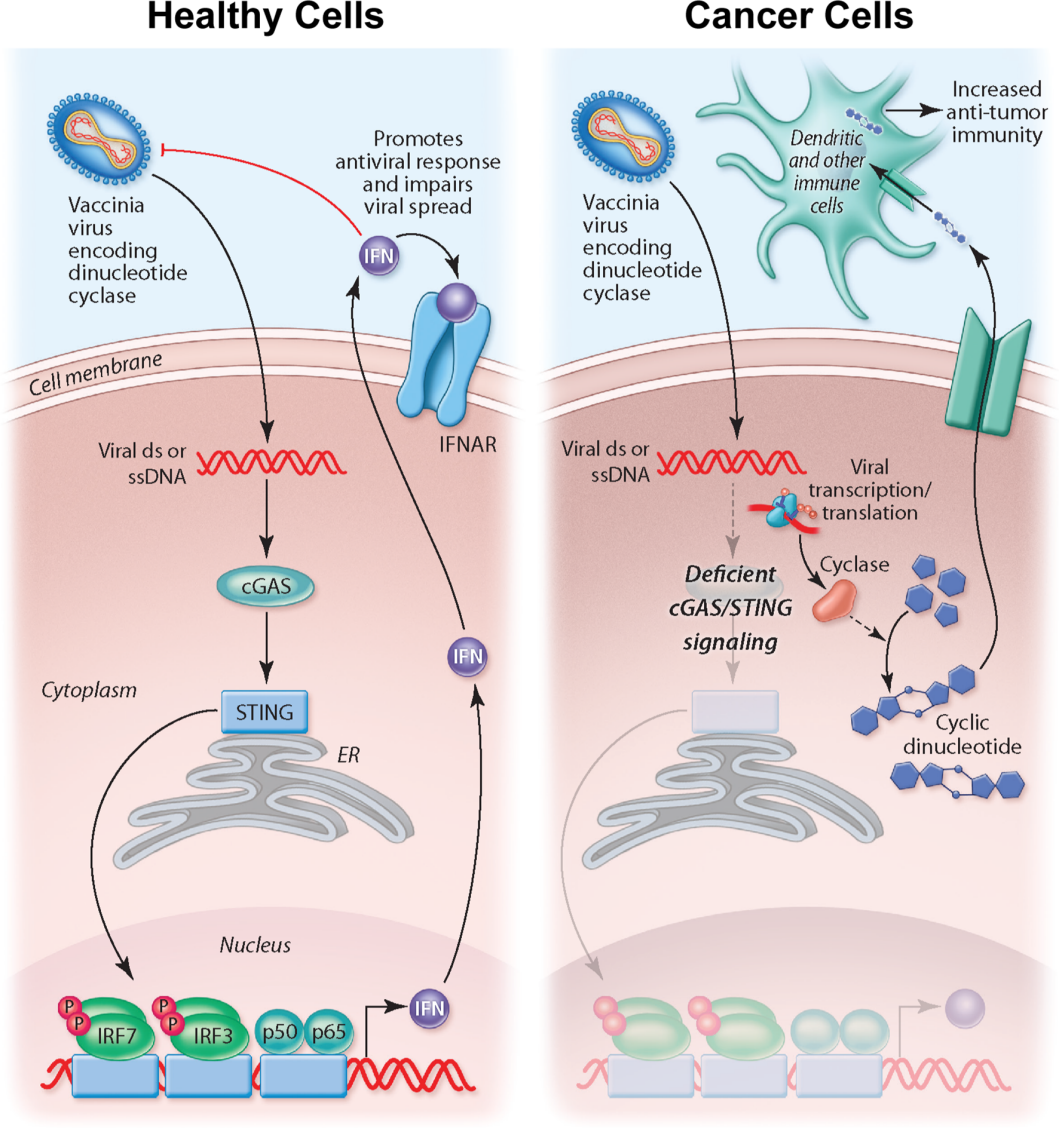
Overview of working model for STINGPOX’s therapeutic effects. In healthy cells, STING signaling is active, and STINGPOX replication would be significantly hindered due to increased activation of IFN signaling. In several cancer types, immune signaling, and more specifically, STING signaling is deficient; therefore STINGPOX-induced cyclic di-AMP production does not enhance IFN signaling/production in infected cells enabling viral replication and expression of disA. disA catalyzes synthesis of c-di-AMP. Through lysis or secretion, c-di-AMP is transferred to extrinsic phagocytic cells, including dendritic cells, which help promote an anti-tumor response.

The importance of STING signaling is highlighted by the very common de-regulation of this pathway in the majority of human cancer cell lines likely enabling tumor escape of immunosurveillance (68–70). We demonstrate that the production of cyclic dinucleotides by the virally encoded disA enzyme not only impacts STING signaling within the infected cell but may also have a bystander impact as agonists are released from infected cells, likely following cell lysis. Indeed, recent work suggests driving cyclic dinucleotide uptake in bystander immune cells and nonhematopoietic cells contributes to the success of STING agonist-based therapeutics (71, 72). Tumor-derived cyclic dinucleotides can be transferred to bystander stromal cells to drive STING signaling in these neighboring cell compartment (72). In particular, driving STING signaling in dendritic cells appears critical to anti-tumor immunity. Therefore, our working model for STINGPOX’s therapeutic effects is that STING-deficient cancer cells have increased susceptibility to the immunogenic virus and serve as a site for c-di-AMP production (see working model summarized in **Figure 6**). While CDNs have minimal immunological influence in STING-deficient cancer cells, through secretion or cell lysis during viral infection, c-di-AMP is released and ingested by extrinsic phagocytes (e.g dendritic cells), activating the STING pathway and promoting anti-tumor immunity (73).

Recent studies have suggested that c-di-AMP may also interact with another membrane adaptor protein (ERAdP) to initiate anti-bacterial immune signaling through the TAK1-NF-κB pathway; future work will examine the relative contributions of ERAdP and STING to the anti-cancer effects of STINGPOX (74). While our study has focused on the application of STINGPOX in treating cancer models, encoding a dinucleotide cyclase in poxvirus vaccine vectors could be useful in the generation of an immune response against an encoded antigen. STING agonists are already being explored for co-administration with vaccines as adjuvants (75). We predict that encoding DisA in a viral vector will have adjuvant-like effects, similar to what has been reported for the BCG vaccine (54).

Extensions of the CRISPR-mediated approach to recombinant poxvirus vector generation should enable rapid arrayed and pooled screening for identification of novel poxvirus-vectored vaccine or oncolytic virus candidates. Similar arrayed screening approaches could also enable interrogation of basic poxvirus biology through the deletion of genomic regions or targeted insertion of various mutant sequences. The feasibility of applying this approach towards screens is reinforced by the decreasing cost of synthetic guide RNA and gene synthesis. This could provide researchers a powerful tool to facilitate the identification of lead candidates given the wide array of transgenes with potential to enhance the efficacy of cancer immunotherapies and vaccines. In the context of oncolytic VACV, vectors encoding a wide variety of classes of transgenes have been developed, including co-stimulatory molecules, cytokines, neoantigens, T-cell engagers, and checkpoint inhibitors [reviewed in (2)]. Direct comparisons of larger groups of these transgenes, or combinations thereof, would facilitate more efficient identification of optimal candidates for clinical translation.

Furthermore, the recent coronavirus virus outbreak worldwide has demonstrated the need for more accessible approaches to accelerate vaccine generation as part of a rapid response (76). Several VACV-vectored vaccines are already in clinical development or approved for use against several infectious diseases, including monkeypox virus, Ebola virus, measles virus, rabies viruses, and respiratory syncytial virus (77). The approach described in this study will enable the accelerated development and screening of additional VACV-based vaccine candidates expressing an array of various antigens and immune modulators. In particular, the described approach could be useful in the rapid generation of personalized cancer vaccines: after identification of patient-specific tumor antigens, the sequences can be rapidly incorporated into a poxvirus-vectored cancer vaccine using the CARVE system. Overall, our work should facilitate the rapid development of poxvirus-vectored vaccines with a wider array of functionality, and enhance the capacity of labs to screen multiple poxvirus-vectored candidates.

## Data availability statement

The original contributions presented in the study are included in the main text or supplementary files. RNA-Seq data presented in the study are deposited in the Gene Expression Omnibus repository, accession number GSE214751. Further inquiries can be directed to the corresponding author.

## Ethics statement

All animal experiments were reviewed and approved by either the University of Ottawa Animal Care and Veterinary Services (MEe-2258-R5) or the Institutional Animal Research Ethics Boards of McMaster University. All research complies with all relevant ethical regulations at OHRI, McMaster University, and the University of Ottawa (biohazardous material use certificate GC317-125-12). Ovarian tumor samples were obtained from cancer patients through the Global Tissue Consenting committee at the OHRI. Patients provided their written informed consent to participate in this study. All animal studies followed the guidelines of the National Institutes of Health and the Canadian Council on Animal care.

## Author contributions

RS, BL, JB, and CI conceived the study. RS, JB, BL and CI supervised the study. RS, JW, BL, CI, and JB wrote the first draft of the manuscript. RS, JW, FW, AP, LT, GP, NM, MC, JP, BA, XH, RM, JD, CJ, EEFF, NA, AC, SB, MH, MT, ZT, and TA contributed to the design of experiments, performing experiments, and/or analysis of data. All authors contributed to the article and approved the submitted version.
