# Supplementary material for: CRISPR-mediated rapid arming of poxvirus vectors enables facile generation of the novel immunotherapeutic STINGPOX
Source: Front Immunol. 2023 Jan 13;13:1050250. doi: 10.3389/fimmu.2022.1050250 (PMC9880309; doi:10.3389/fimmu.2022.1050250)
Supplement: Supplementary file 1 [file DataSheet_1.docx]

***Supplementary Material***

**CRISPR-mediated rapid arming of poxvirus vectors enables facile generation of the novel immunotherapeutic STINGPOX**

Authors

Jack T Whelan^1,2†,^, Ragunath Singaravelu^1,2,3†^, Fuan Wang^4,5†^, Adrian Pelin^1,2†^, Levi A Tamming^1,2^, Giuseppe Pugliese^2,^, Nikolas T Martin^1,2,^, Mathieu JF Crupi^1,2,^, Julia Petryk^2,^, Bradley Austin^2,^, Xiaohong He^2,^, Ricardo Marius^1,2,^, Jessie Duong^1,2,^, Carter Jones^2,^, Emily EF Fekete^1,2^, Nouf Alluqmani^1,2^, Andrew Chen^2,^, Stephen Boulton^1,2,^, Michael S. Huh^2,^, Matt Y. Tang^2,^, Zaid Taha^1,2^, Elena Scut^1,2^, Jean-Simon Diallo^1,2,^, Taha Azad^1,2,^, Brian D Lichty^4,5,^*, Carolina S Ilkow^1,2,^*, John C Bell^1,2,^*

Affiliations

^1^Department of Biochemistry, Microbiology and Immunology, University of Ottawa, Ottawa, Ontario, Canada.

^2^Centre for Innovation Cancer Therapeutics, Ottawa Hospital Research Institute, Ottawa, Ontario, Canada

^3^Public Health Agency of Canada, Ottawa, Ontario, Canada

^4^McMaster Immunology Research Centre, Department of Medicine, McMaster University, Hamilton, Ontario, Canada

^5^MG DeGroote Institute for Infectious Disease Research, McMaster University, Hamilton, Ontario, Canada

^†^These authors have contributed equally to this work and share first authorship.

*Correspondence:

John C. Bell, Center for Innovation Cancer Therapeutics, Ottawa Hospital Research Institute, Ottawa, ON K1H 8L6, Canada. E-mail: [jbell@ohri.ca](mailto:jbell@ohri.ca)

Carolina S. Ilkow, Center for Innovation Cancer Therapeutics, Ottawa Hospital Research Institute, Ottawa, ON K1H 8L6, Canada. E-mail: [cilkow@ohri.ca](mailto:cilkow@ohri.ca)

Brian D. Lichty, McMaster Immunology Research Centre, Department of Pathology and Molecular Medicine, McMaster University, Hamilton, ON L8N 3Z5, Canada. E-mail: [lichtyb@mcmaster.ca](mailto:lichtyb@mcmaster.ca)

**Supplementary Table 1 |** List of oligonucleotides and sgRNAs used in this study

| **Primer Name** | **Sequence** |
| --- | --- |
| **BamHI-Cas9-F** | ATGGATCCGCCACCATGGACAAGAAGTAC |
| **Cas9-XbaI-R** | ATTCTAGATTAGTCGCCTCCCAGCTGAG |
| **sgB8R_oligo_F** | CACCGAGAATTATAATATATCTCA |
| **sgB8R_oligo_R** | AAACTGAGATATATTATAATTCTC |
| **sgNTC_oligo_F** | CACCGGCGAGGTATTCGGCTCCGCG |
| **sgNTC_oligo_R** | AAACCGCGGAGCCGAATACCTCGCC |
| **qhRSAD2 - F** | GAGAAGCACAACAGGAGAGCAA |
| **qhRSAD2 - R** | GAAGTGATAGTTGACGCTGGTT |
| **qhIFNB1 - F** | TCTGGCACAACAGGTACTAGGC |
| **qhIFNB1 - R** | GAGAAGCACAACAGGAGAGCAA |
| **q18S rRNA - F** | GCGATGCGGCGGCGTTATTC |
| **q18S rRNA - R** | CAATCTGTCAATCCTGTCCGTGTCC |
| **pUC19_F** | AGCTGCATGTGTCAGAGG |
| **pUC19_R** | TATCCACAGAATCAGGGG |
| **pB8R_5’HD_F** | TTCCTGCGTTATCCCCTGATTCTGTGGATAACGCGTGTGTAAAACTCTATATAGATGG |
| **pB8R_5’HD_R** | GGTGAACAGCTCCTCGCCCTTGCTCACCATGGTGTTGTTTGTTATTTGACTAC |
| **pB8R_3’HD_F** | CACTCTCGGCATGGACGAGCTGTACAAGTAGGGTGGCAAACAATGTATACAAATG |
| **pB8R_3’HD_R** | TGACGGTGAAAACCTCTGACACATGCAGCACGCGTTGGATCTAATTGCACATGTATATAC |
| **pB8R_EGFP_F** | ATGGTGAGCAAGGGCG |
| **pB8R_EGFP_R** | CTACTTGTACAGCTCGTCCATG |
| **B8R_HR_F** | TATTCACAAATATGATGGTGATG |
| **B8R_HR_R** | CGCCTTGTTCAAATTTAACCCAG |
| **B8R_Screen_F** | GACTCGTCTACTATTCAACGCAGAGG |
| **B8R_Screen_R** | GTGGCAAAGTCTATGCTGCAACC |
| **J2R_HR_F** | CCCTATTGTTACAGATGGAAGGGTCA |
| **J2R_HR_R** | CTATATACACTACGGTGGCACCATC |
| **J2R_Screen_F** | GCCGTGGGTCATTGTTATGAATCTC |
| **J2R_Screen_R** | AGGATCATGATGGCGTCCGTCA |
| **A46R_HR_F** | CATCTGCACTAATTAACAGTTTTACTATATACCTGC |
| **A46R_HR_R** | ACAAGATGAGCTACTCATCGGTGATG |
| **A46R_Screen_F** | CATCTGCACTAATTAACAGTTTTACTATATACCTGC |
| **A46R_Screen_R** | ACAAGATGAGCTACTCATCGGTGATG |
| **I4L_HR_F** | CGGGCGACAGATGCTAGC |
| **I4L_HR_R** | CGCTGGTCCAGAACTGATACC |
| **I4L_Screen_F** | TTTCGGAATCGCAAACCACCAG |
| **I4L_Screen_R** | GGTAATTTCTGGCGCCGCC |
| **sgB8R*** | GAGAAUUAUAAUAUAUCUCA |
| **sgJ2R*** | UGUUGAAUUCUGUGAGCGUA |
| **sgA46R*** | ACAAGUAAGUCAUACUAAC |
| **sgI4L*** | GUUAUUCAAGGAAUAUAUAA |

*Sequence reflects only targeting sequence for sgRNA

**Supplementary Table 2 |** Protein coding sequences and primary product for cyclases screened in this study

| **Cyclase** | **NCBI Reference or Genbank sequence** | **Primary CDN product** |
| --- | --- | --- |
| disA | WP_003900111 | 3’3’-c-di-AMP |
| cdaA | WP_001052610 | 3’-3’-c-di-GMP |
| VCA0848 | WP_000998604 | 3’-3’-c-di-GMP |
| VC2285 | Q9KPS7 | 3’-3’-c-di-GMP |
| PA2771 | WP_033956084 | 3’-3’-c-di-GMP |
| PA3702 | WP_106451200 | 3’-3’-c-di-GMP |
| ECSP_2022 | WP_000592852 | 3’-3’-c-di-GMP |

**Supplementary Table 3 |** Gene ontology analysis classifying genes activated >4 fold during STINGPOX infection relative to OncoVACV infection in primary human macrophages (MOI 3; 18h) by biological processes*

| **ID** | **Name** | **P value**** |
| --- | --- | --- |
| GO:0009615 | response to virus | 6.04E-13 |
| GO:0019221 | cytokine-mediated signaling pathway | 2.74E-12 |
| GO:1903706 | regulation of hemopoiesis | 5.08E-11 |
| GO:0034097 | response to cytokine | 8.21E-10 |
| GO:0051607 | defense response to virus | 2.22E-09 |
| GO:0140546 | defense response to symbiont | 2.32E-09 |
| GO:0071345 | cellular response to cytokine stimulus | 3.14E-09 |
| GO:0045637 | regulation of myeloid cell differentiation | 2.42E-08 |
| GO:0045087 | innate immune response | 2.56E-08 |
| GO:0098542 | defense response to other organism | 2.65E-08 |

***Only top 10 overrepresented pathways are listed.**

****Adjusted with Bonferroni correction**


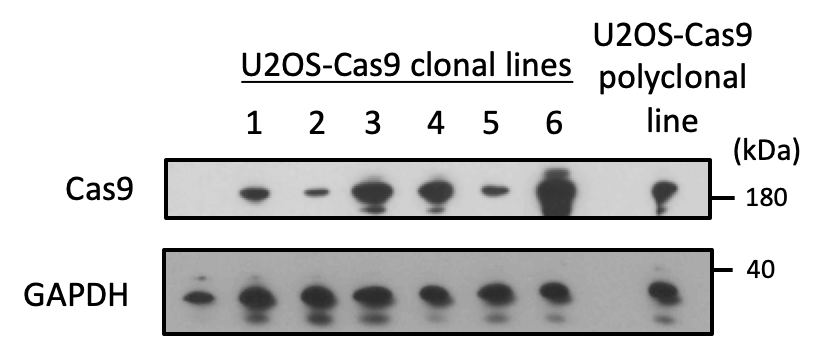


**Supplementary Figure 1 |** Whole cell lysates of clonal populations of U2OS cells transduced with lentivirus expressing Cas9 (without NLS) were analyzed by Western blot using an anti-Cas9 antibody to screen for highest-expressing population. Clone 6 was selected for use in the remainder of the study. GAPDH levels were probed as a loading control.


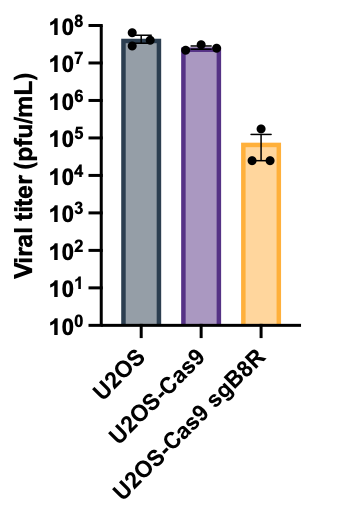
**Supplementary Figure 2 |** B8R targeted sgRNA/Cas9 complex impairs VACV replication. U2OS cell lines stably expressing Cas9, Cas9 and a guide RNA targeting the B8R locus, or wildtype cells were infected with Copenhagen strain of VACV for 72 hours (MOI = 0.01), and then plaque assays were performed to measure virus production in supernatants.


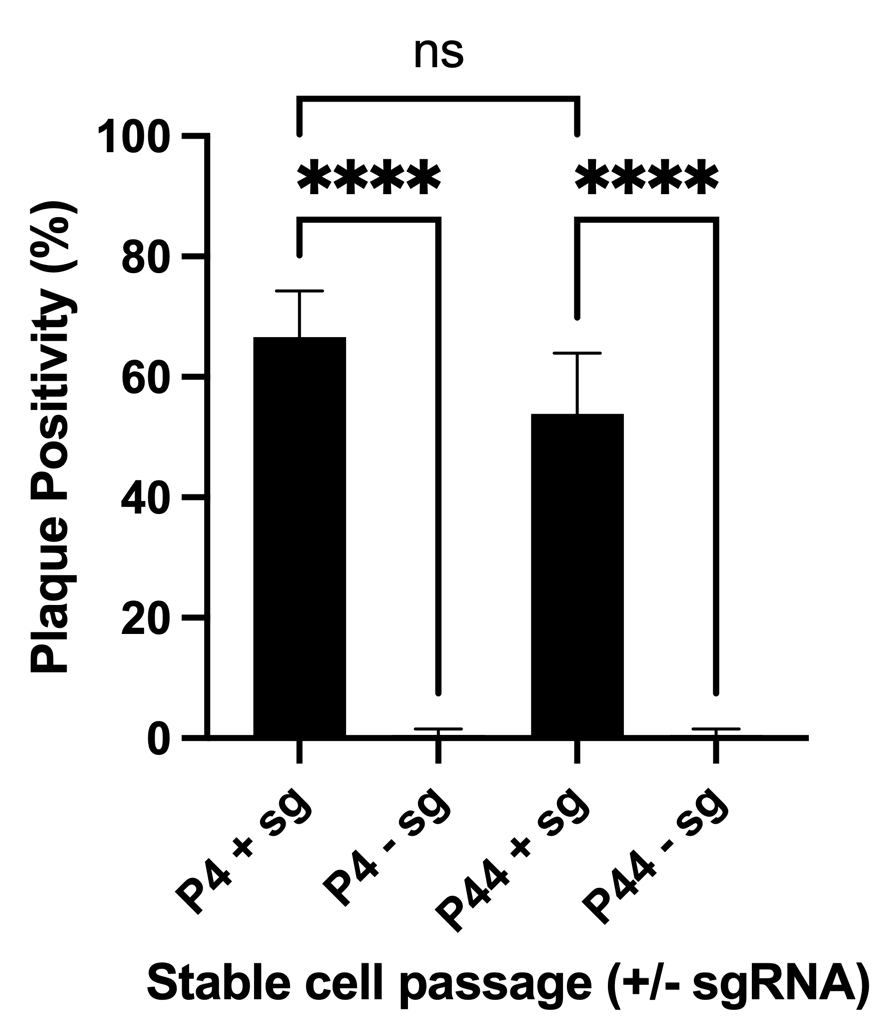


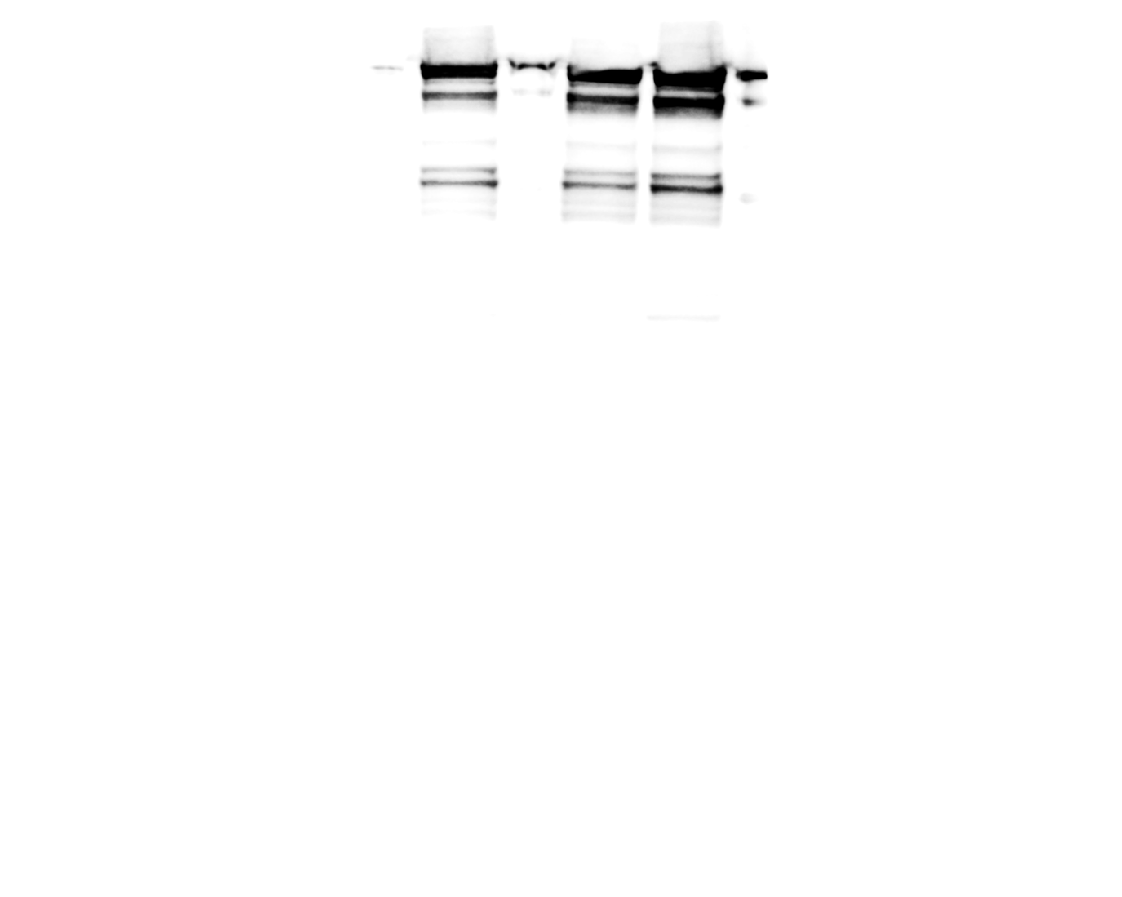

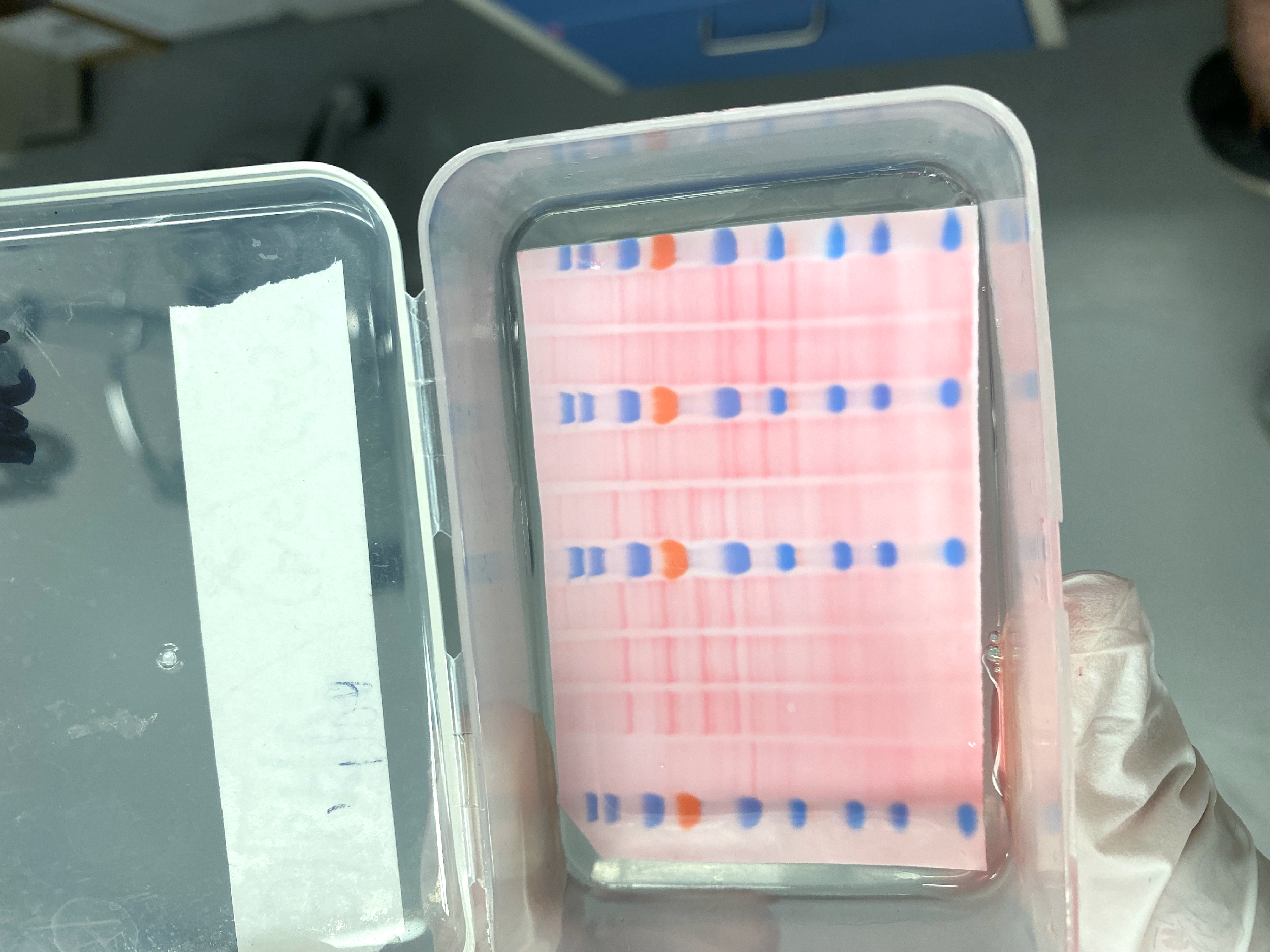


Cas9

Ponceau

P+4

P+44

140 kDa

140 kDa

65 kDa

**A**

**Supplementary Figure 3 |** **A.** Whole cell lysates of U2OS-Cas9 stable cells were analyzed by Western blot using an anti-Cas9 antibody confirming durable expression of Cas9. Ponceau stain was included to ensure equal loading. **B.** U2OS-Cas9 cells at passage +4 and passage +44 from clonal isolation were used to facilitate the recombination of VACV at the B8R locus. Resulting progeny virus was used to inoculate U2OS monolayers and scored for inclusion of EGFP at B8R loci (n =3). (p**** < 0.001).


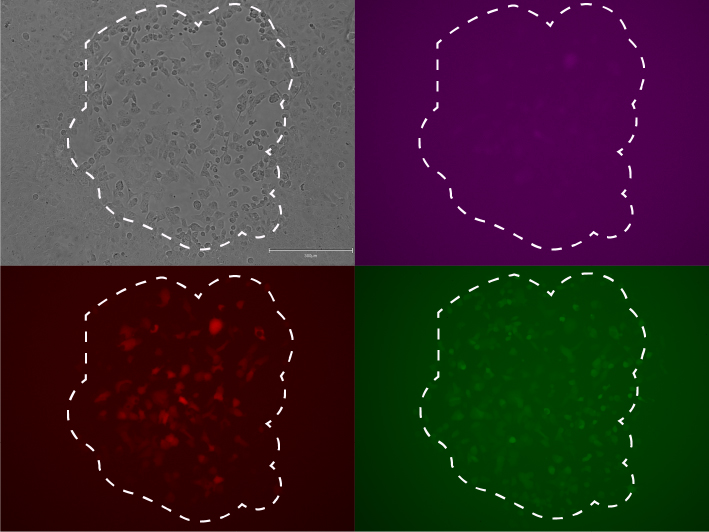


A

B

D

C

**Supplementary Figure 4** | Fluorescent microscopy of Cop -B8R-TK-A46R. Single plaque (A) of Copenhagen VACV expressing iRFP720 at the A46R loci (B) mCherry at the TK loci (B) and EGFP at the B8R loci (D). Imaged at 100X magnification, scalebar is proportionate to 300 μM.
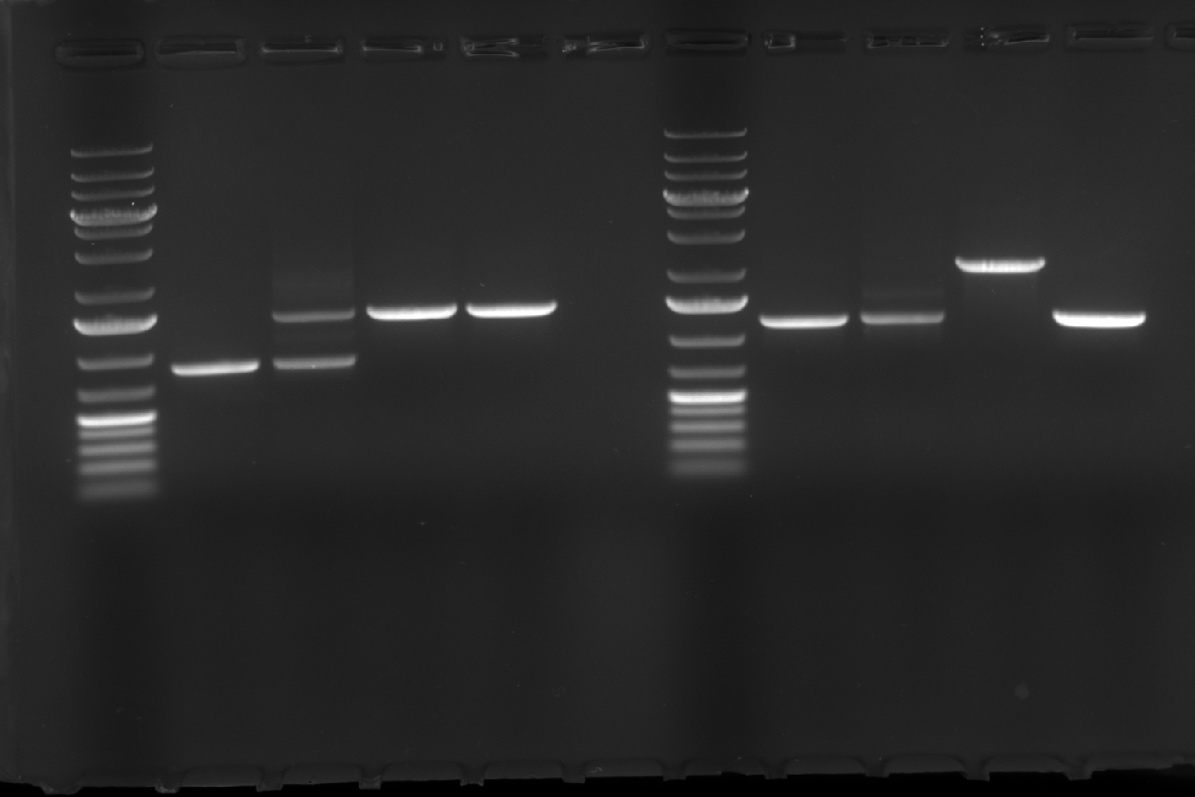


Cop -A46R mixed

Cop WT

Cop -B8R-J2R-A46R

Cop -B8R-I4L-A46R

1690 bp

2158 bp


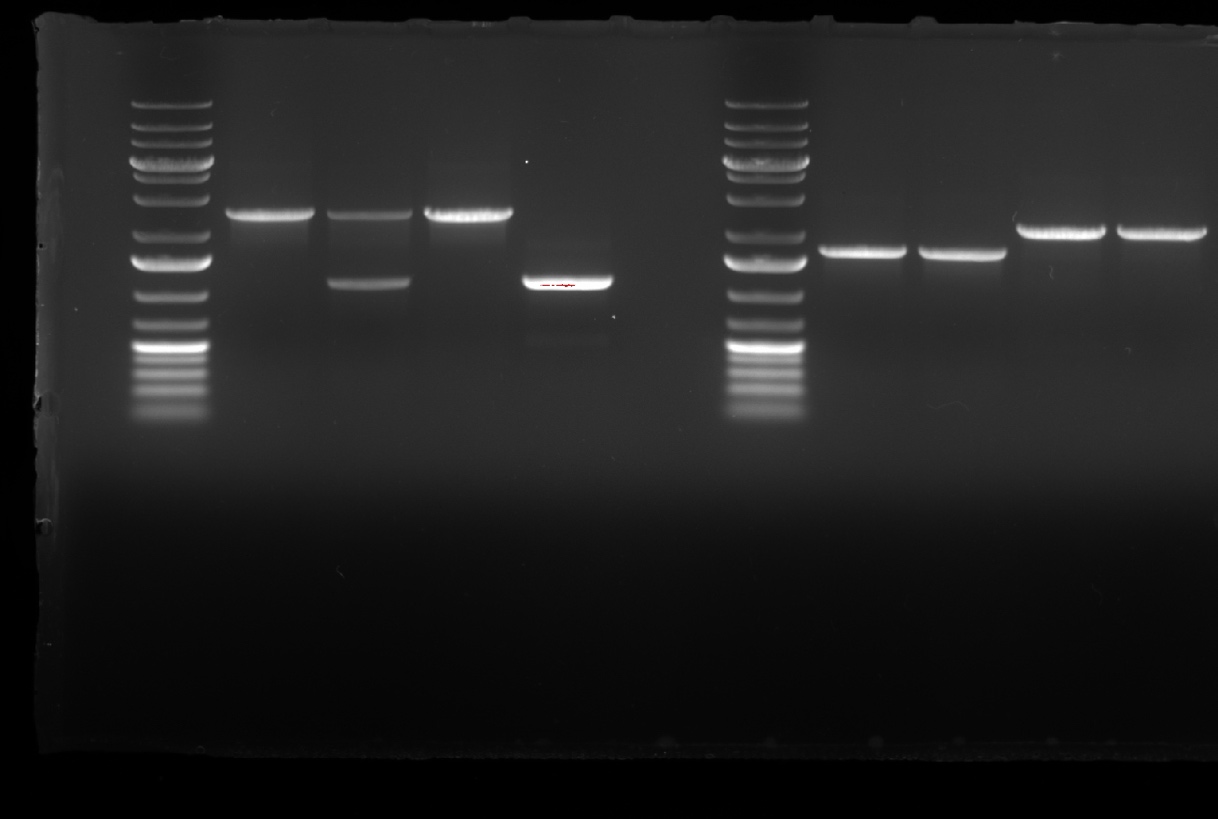


Cop -I4L mixed

Cop WT

Cop -B8R-J2R-A46R

Cop -B8R-I4L-A46R

981 bp

2359 bp


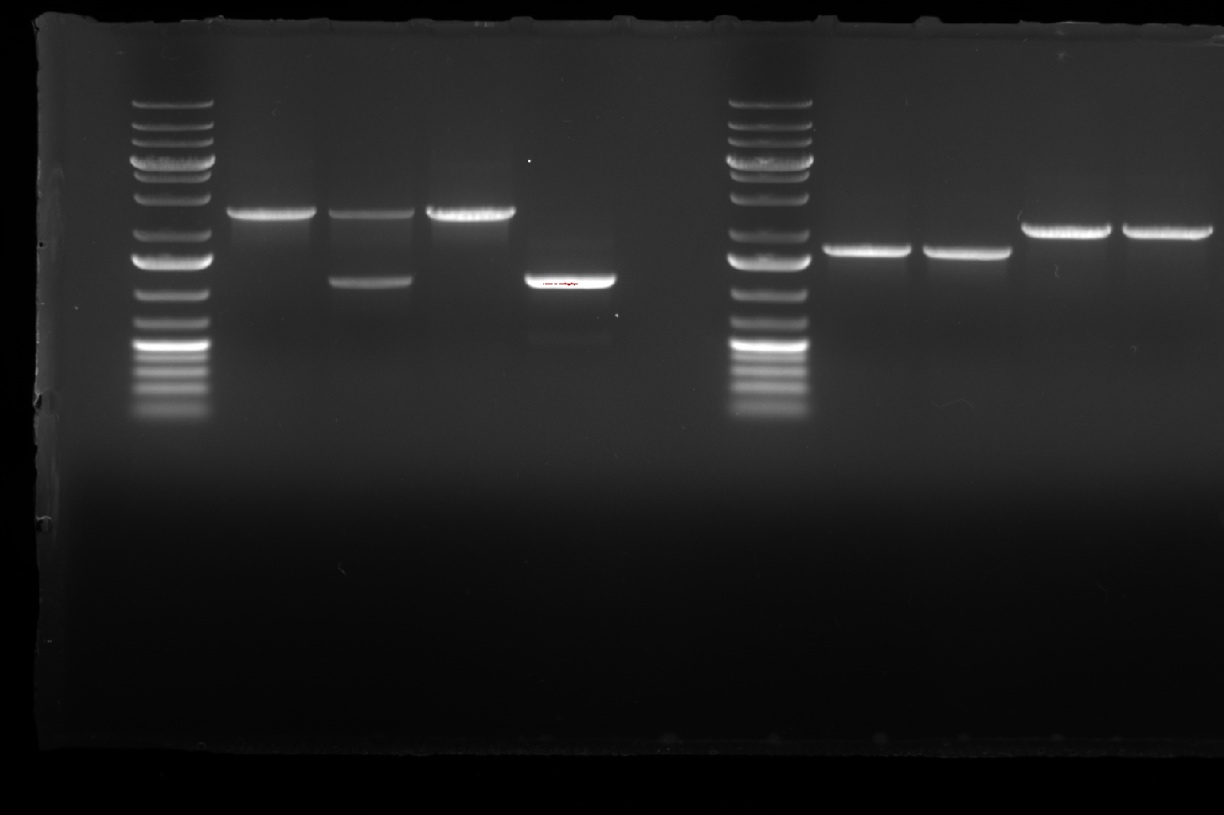


Cop – J2R mixed

Cop WT

Cop -B8R-J2R-A46R

Cop -B8R-I4L-A46R

1211 bp

2104 bp

Cop -B8R mixed

Cop WT

Cop -B8R-J2R-A46R

Cop -B8R-I4L-A46R

869 bp

1584 bp


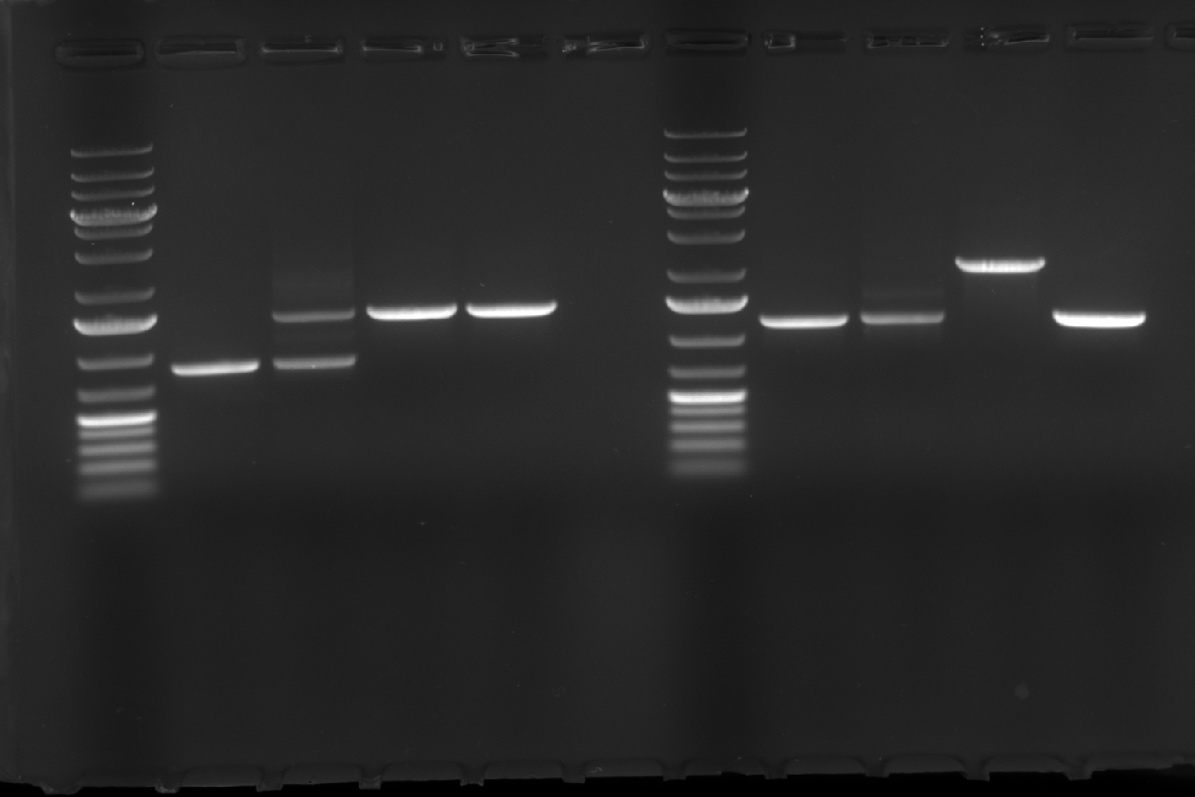


**D**

**B**

**A**

**C**

**Supplementary Figure 5** | PCR Screening of isolated plaques for Copenhagen with three CARVE-mediated insertions. Screening primers flanking B8R (A), J2R (B), I4L (C), and A46R (D) insertion sites within the Copenhagen genome were designed for each loci yielding different sized PCR fragments following insertion of HRT sequence (Supplementary Table 1). Results for Cop -B8R-J2R-A46R and Cop -B8R-I4L-A46R are representative of 16 plaques isolated in a second round following CARVE protocol. Results of plaques were compared to wild-type Copenhagen (Cop WT) as well as mixed first-round populations from of CARVE protocol as controls.


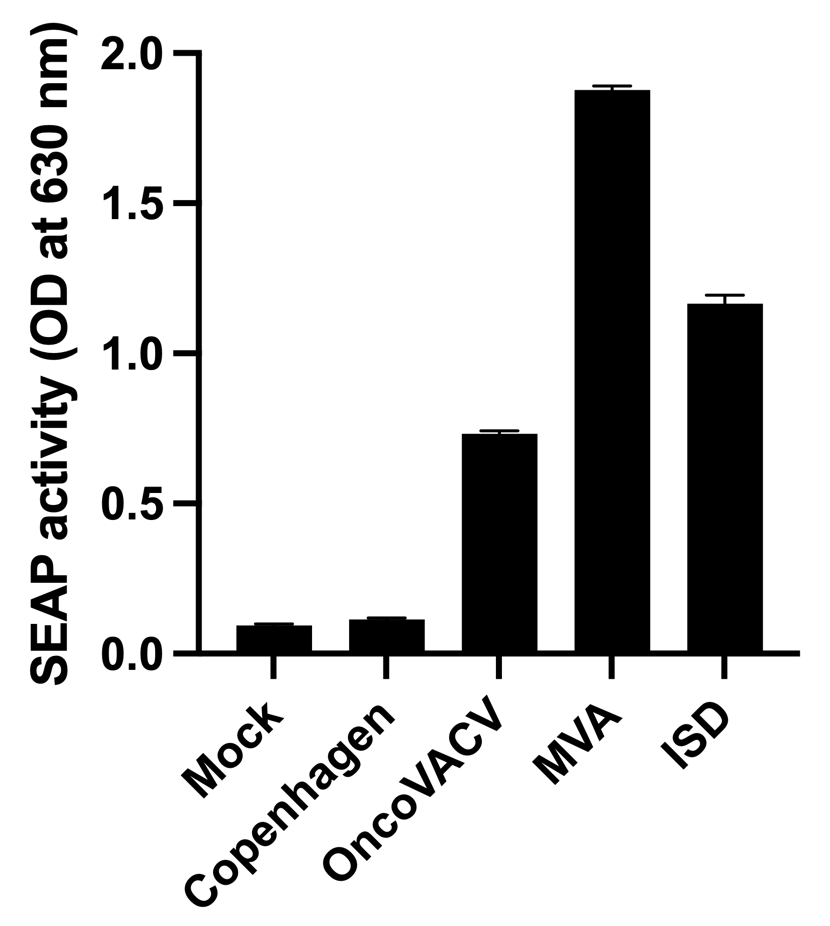


**Supplementary Figure 6 |** MVA infection drives increased IFN signaling in comparison to OncoVACV**.** THP1-Blue-ISG cells were infected with various vaccinia viruses (MVA, OncoVACV, or Copenhagen; MOI = 5). 24 hours post-infection, interferon signaling was monitored via SEAP assay and detection of OD at 630 nm. Model cytosolic dsDNA, immunostimulatory DNA (ISD), was used as a positive control.

**
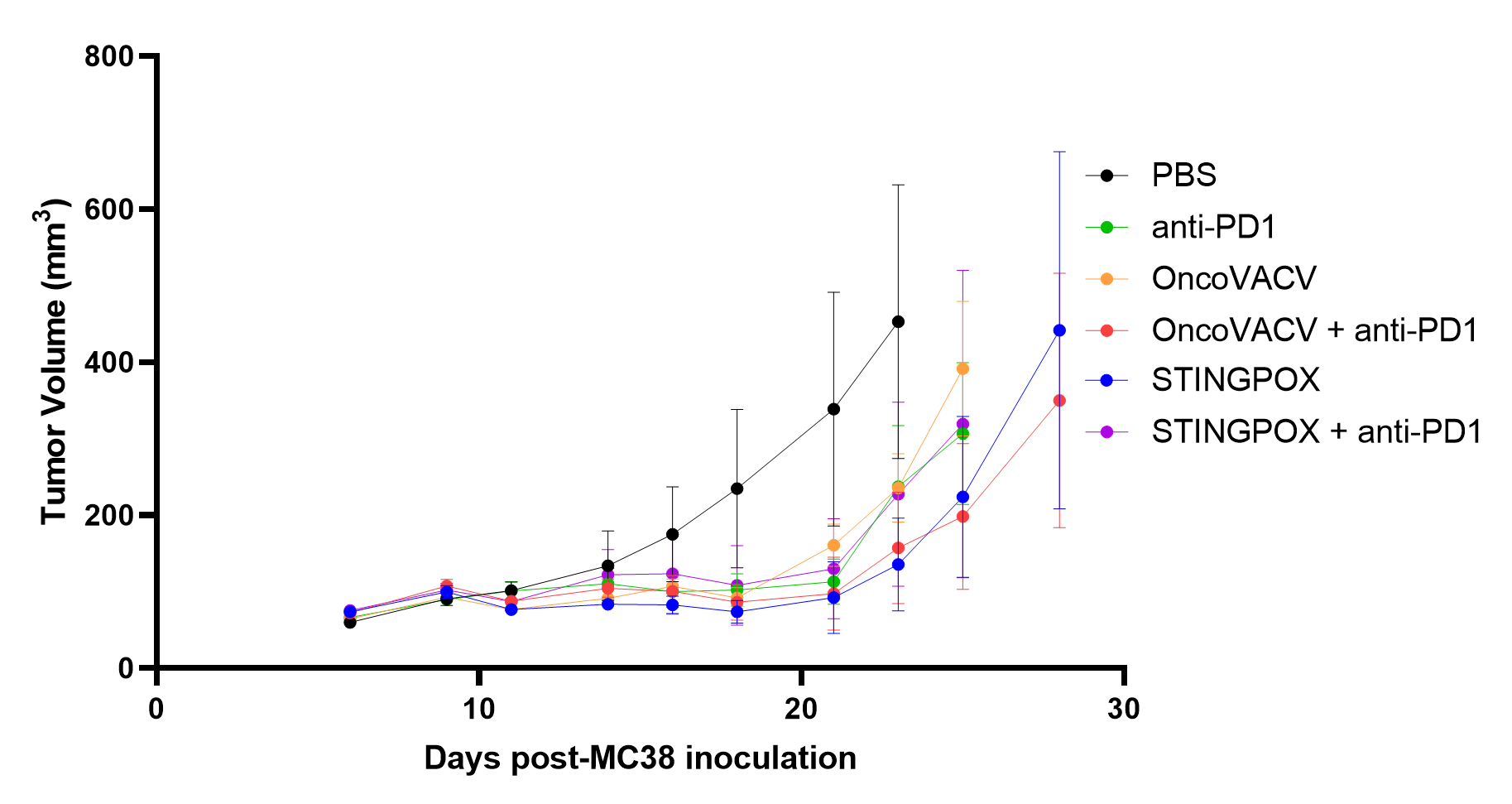
**

**Supplementary Figure 7 |** STINGPOX induces anti-tumor effects in vivo. Mice bearing subcutaneous MC38 colorectal tumors were treated with 3 doses intratumorally of PBS, OncoVACV, or STINGPOX and anti-PD1 as indicated in the timeline in Figure 5B. Mean tumor volume curves are shown with error bars representing SEM (n=10).

**
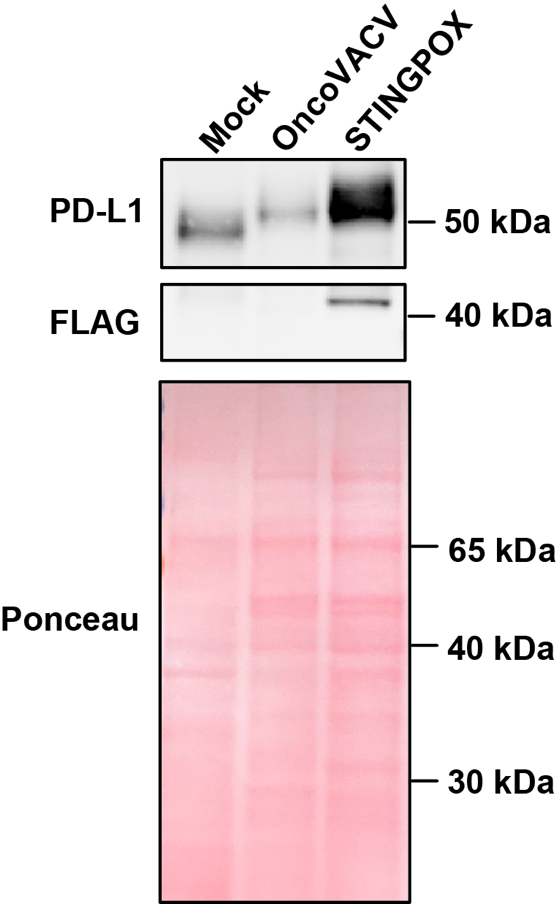
**

**Supplementary Figure 8** | B16-OVA cells were infected *in vitro* with OncoVACV-GFP and STINGPOX (MOI = 1), and 48 hours post-infection, whole cell lysates were analyzed by WB for PD-L1 and disA (FLAG) expression. Ponceau staining of blot is shown for loading control.


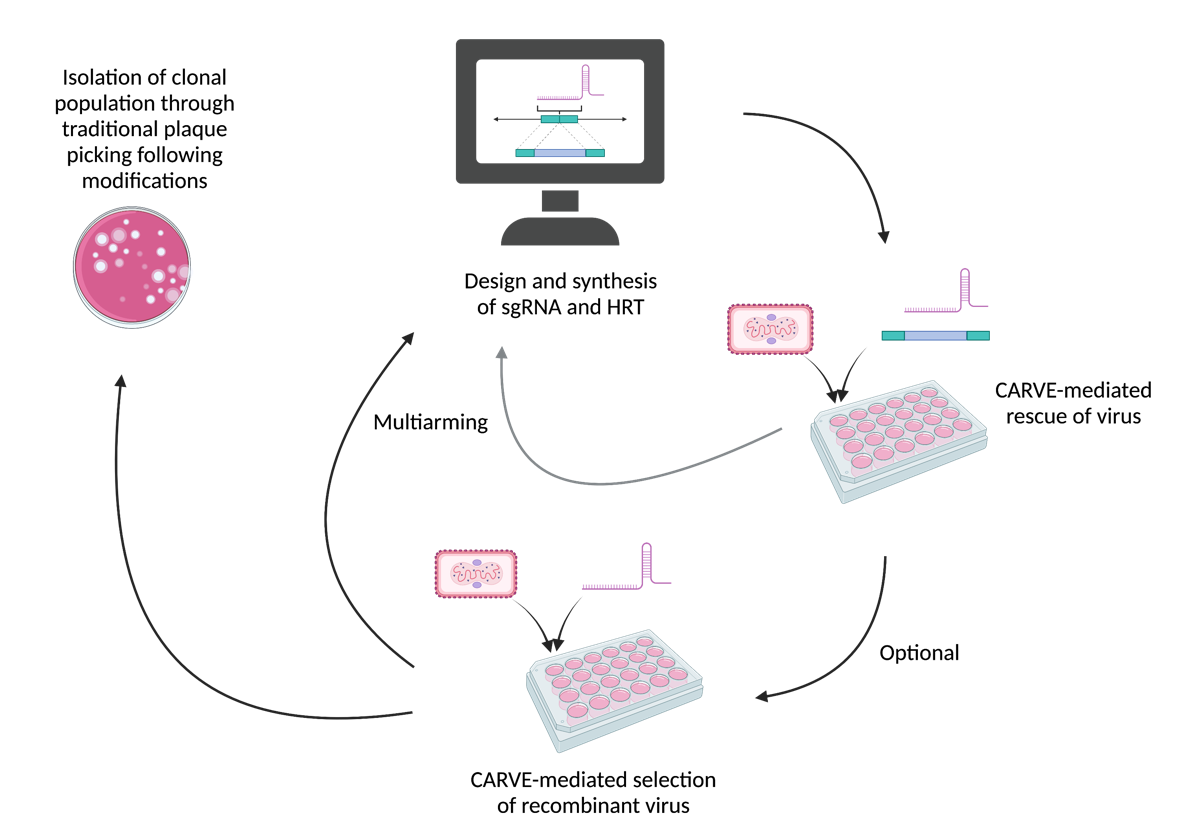


**Supplementary Figure 9** | Outline of CARVE system from design to isolation. A single guide RNA (sgRNA) targeting the loci of interest can be designed alongside our homologous recombination template (HRT) and ordered for synthesis. Following their synthesis, the sgRNA and HRT can be used in an infection/transfection to facilitate the recombination of your poxvirus backbone. Further selection or multi-arming can be undertaken if necessary for your project goals, before the isolation of your pure viral population through plaque purification or similar clonal isolation methods. Figure created with BioRender.com.
